# The development and feasibility of a personal health-optimization system for people with bipolar disorder

**DOI:** 10.1186/s12911-017-0481-x

**Published:** 2017-07-10

**Authors:** Øystein Eiring, Kari Nytrøen, Simone Kienlin, Soudabeh Khodambashi, Magne Nylenna

**Affiliations:** 1 Faculty of Medicine, University of Oslo, Postbox 1072, Blindern, N-0316 Oslo Norway; 2 Norwegian Institute of Public Health, Postbox 4404, Nydalen, N-0403 Oslo Norway; 3 Department of Medicine and Healthcare, South-Eastern Norway Regional Health Authority, Postbox 404, N-2303 Hamar, Norway; 4 Oslo University Hospital, Postbox 4950, Nydalen, N-0424 Oslo Norway; 5 Department of Medicine, University Hospital of North Norway, Postbox 6050, N-9037 Langnes, Tromsø, Norway; 6 Norwegian University of Science and Technology, N-7491 Trondheim, Norway

**Keywords:** Shared decision-making, Patient decision aid, Clinical decision support system, Patient participation tool, Compliance, Adherence, E-health, M-health, Bipolar disorder, Clinical practice guideline

## Abstract

**Background:**

People with bipolar disorder often experience ill health and have considerably reduced life expectancies. Suboptimal treatment is common and includes a lack of effective medicines, overtreatment, and non-adherence to medical interventions and lifestyle measures. E- and m-health applications support patients in optimizing their treatment but often exhibit conceptual and technical shortcomings. The objective of this work was to develop and test the usability of a system targeting suboptimal treatment and compare the service to other genres and strategies.

**Methods:**

Based on the frameworks of shared decision-making, multi-criteria decision analysis, and single-subject research design, we interviewed potential users, reviewed research and current approaches, and created a first version using a rapid prototyping framework. We then iteratively improved and expanded the service based on formative usability testing with patients, healthcare providers, and laypeople from Norway, the UK, and Ukraine. The evidence-based health-optimization system was developed using systematic methods. The System Usability Scale and a questionnaire were administered in formative and summative tests. A comparison of the system to current standards for clinical practice guidelines and patient decision aids was performed.

**Results:**

Seventy-eight potential users identified 82 issues. Driven by user feedback, the limited first version was developed into a more comprehensive system. The current version encompasses 21 integrated core features, supporting 6 health-optimization strategies. One crucial feature enables patients and clinicians to explore the likely value of treatments based on mathematical integration of self-reported and research data and the patient’s preferences. The mean ± SD (median) system usability score of the patient-oriented subsystem was 71 ± 18 (73). The mean ± SD (median) system usability score in the summative usability testing was 78 ± 18 (75), well above the norm score of 68. Feedback from the questionnaire was generally positive. Eighteen out of 23 components in the system are not required in international standards for patient decision aids and clinical practice guidelines.

**Conclusion:**

We have developed the first evidence-based health-optimization system enabling patients, clinicians, and caregivers to collaborate in optimizing the patient’s health on a shared platform. User tests indicate that the feasibility of the system is acceptable.

**Electronic supplementary material:**

The online version of this article (doi:10.1186/s12911-017-0481-x) contains supplementary material, which is available to authorized users.

## Background

Bipolar disorder is a leading cause of poor health worldwide, with a lifetime prevalence of 2.4% [1]. The results of treatment are often suboptimal: 60–85% of patients receiving treatment experience at least one manic or depressive relapse in 4–5 years, and residual symptoms, functional disability, and cognitive impairment between relapses are common [2]. At least one third of patients do not respond to their treatment [3]. Poor adherence to medication affects 20–60% of patients [4] and is associated with hospitalizations, recurrent mood episodes, and an increased risk of suicide attempts [5]. The deficit in adherence is comparable to that seen for other common chronic conditions, which is estimated to range between 35–72% [6]. The symptoms of the disease and the effects of medicines can vary by patient and over time, and they are often unpredictable. For many patients, treatment based on trial and error is unavoidable.

*Medicine optimization* is a framework targeting many of the reasons for suboptimal treatment. The approach is defined as “a person-centered approach to safe and effective medicine use to ensure people obtain the best possible outcomes from their medicines.” [7].

It should be supported by appropriate tools, such as patient decision aids; clinical decision support; and self-management, monitoring, and communication systems [7]. Medicine optimization integrates the patient’s values and preferences with the best available evidence, measurement of outcomes, and shared decision-making [8].

Importantly, none of the technologies deemed essential to medicine optimization have unequivocally been shown to improve patient health. Patient decision aids can increase general knowledge, enhance the proportion of patients that select a treatment consistent with their values, foster more involvement, and improve communication [9]. However, no significant effect on health outcomes has been found. Clinical decision support systems might improve morbidity outcomes and inappropriate prescriptions but do not significantly reduce adverse events or mortality [10–12]. Electronic reminders can improve short-term medication adherence, but 61% of m-health chronic disease management systems and adherence tools do not significantly improve clinical outcomes [13, 14].

One reason for these modest health benefits might be that the technologies exist in separate silos: one for patients and a different one for clinicians. Systems involving both the patient and the clinician are more likely to succeed [15], but only a small minority of decision support systems include patient decision aids, and even fewer allow patients and physicians to jointly interact with the system [16]. To adequately inform clinicians, systems should include real-time monitoring and evaluation [17].

Current prescription guidelines provide additional requirements for a merged system; the information must be evidence-based, and contraindications and potential adverse effects must be considered. Outcomes important to the individual patient should guide the assessment of treatments, and systematic monitoring and review should be facilitated [18, 19]. Adherence, symptoms, and other medical data should be monitored, enabling treatment assessments and decisions to be informed by longitudinal data [20].

Suboptimal medicine use is not the only reason bipolar disorder patients have life expectancies 10 to 20 years shorter than the general population. Other prominent factors are poor lifestyle behaviors, including poor diet, a lack of exercise, and smoking. A lack of coordinated care and management and limited social support also figure among the causes [21]. To holistically support the optimization of treatment, an application should therefore focus on medicines and other interventions, such as a healthy lifestyle and social support.

The aim of this paper is to describe the development and usability testing of a comprehensive health-optimization system for patients with bipolar disorder.

## Methods

### Theoretical frameworks

In shared decision-making [22], patients and healthcare personnel weigh the pros and cons of treatment options and try to find the option most compatible with the patient’s personal preferences [22, 23]. The main principles of shared decision-making are operationalized in and supported by patient decision aids, a broadly defined tool expected to comply with more than 40 internationally agreed upon requirements [24, 25].

The second framework applied in this research was multi-criteria decision analysis (MCDA) [26], a sub-discipline of operations research. MCDA approaches are increasingly being used to optimize healthcare decisions [27].

MCDA partly overlaps with shared decision-making. An MCDA-based approach that has been extensively researched was the original conceptual and practical stimulus for this work [28–31]. The MCDA framework was used in design, to model the system database, to integrate input from multiple sources consistently, and to connect the main system modules into a coherent whole.

Single-subject research designs (SSRD) are used to establish whether a treatment is effective for an individual rather than for a group [32] and served as a third framework. This type of design enables patients and clinicians to minimize the element of trial and error in finding the optimal treatment and ensures that this process is systematic and effective. Two main tools within SSRD, also called “*n* = 1 trials,” are visual inspection and statistical analysis tools. As treatments are added or removed, visual inspection of graphs allows the viewers to gauge the effects of the changes on the patient’s health. More than 400 studies have been conducted using SSRDs [33].

### Requirements

The main objective was to create a system enabling long-term follow-up and optimization of treatment and health based on the frameworks. The project was initially a work package in an innovation and research program created to improve suboptimal clinical practices [34].

Formally starting in 2010, the requirement analysis process included interviews and discussions with patients, health professionals, hospital ICT departments, and researchers investigating clinical decision support and patient decision aids. We performed an analysis of personalization in current patient decision aids [35], reviewed existing solutions, performed focus group interviews eliciting users’ needs [36] and reviewed current MCDA-based decision dashboards [37–39].

A detailed analysis and description on how the system should work in clinical practice was developed [40]. In summary, “the system will assist the evaluation, monitoring, selection, and follow-up of pharmacotherapy in chronic disorders. It will be used repeatedly to improve decisions over time and visualize trends in crucial decision components. It will continuously provide opinions about what constitutes the best decision – calculated from continuously updated, personalized evidence, and the individual’s preferences. The system will be designed as a clinical decision support system, a self-management system, and a tool for communication and reflection” (minor amendments made for readability).

In accordance with software requirements used in Norwegian hospitals, the system was constructed and is maintained on Windows Server 2012 R2 using the programming languages C#, JS, and the database Azure SQL Server 2016. For details, see Additional file 1.

The main programmer and the project leader analyzed the initial requirements and constructed a functional design description with a sufficient level of detail for system design. New descriptions were added, discussed, and improved in the project management tool JIRA ® throughout the project (Additional file 2).

As of January 2017, nearly all features of the system pertained to one of the following six health-optimization strategies: Find the best, safe treatment, find the best dosage, increase adherence, live more healthily, get support from professionals and improve the decision process.

Additional file 3 presents the features supporting each strategy.

### Development

#### Information development methods

To develop evidence-based and user-centric descriptions of options, outcomes, expected performances, and background information, we performed a systematic review and conducted focus group interviews with people with bipolar disorder. First, we used patient input to create descriptions of outcomes [36, 41]. Ratings for all treatment options were then calculated from estimates in a network meta-analysis and from surveys conducted by the research team among patients and clinical experts [42]. Descriptions of options included contraindications and were developed from summaries of product characteristics (SPCs) and the drug database MicroMedix. Descriptions of bipolar disorder, the decisions targeted in the system, and shared decision-making incorporated patients’ views from the focus groups, all processes described in detail elsewhere [36]. The texts were reviewed by patients and clinicians and refined in multiple iterations.

### Usability evaluation

#### Participants and settings

From August 2014 to August 2016, 78 potential users participated in one or more usability testing sessions: 39 laypeople, 23 patients, 5 nurses, 2 general practitioners, and 9 psychiatrists. So as not to burden patients unnecessarily and to develop a generic platform applicable for a different conditions and users, features deemed not to be condition-specific were tested with laypeople and identified issues corrected before testing with patients and clinicians.

Patients were recruited from the Facebook page of the Norwegian patient organization and via Bipolar UK. Inclusion criteria for patients were: 1) aged between 18 and 65, 2) a diagnosis of bipolar disorder, and 3) seeing a general practitioner or psychiatrist regularly. Clinicians were recruited from Diakonhjemmet, Innlandet hospital trusts and the primary health care service in Norway. Laypeople were mainly recruited from Facebook and a local college in Norway. From mid-2015, all participants received a gift card after test completion equivalent to USD 40.

### Methods for evaluating usability

#### Formative usability testing

In formative usability tests, qualitative reactions to user interface concepts and designs are obtained [43, 44]. Guided by the conceptual framework of user-centered design, each test was effectuated using a rigorous process that included initially reading aloud a predefined script with information and instructions. Tasks and scenarios relevant for the core functionalities (Additional file 2) were developed by the researchers, designer, and QA personnel and piloted with at least one person [45, 46]. Participants were instructed to think aloud during the tests. Performed with one individual at a time, all tasks were neutrally presented, and no assistance was given. Voice and screen activities were recorded and later analyzed by the team. After the session, we asked the users about any difficulties encountered and whether they had suggestions for improvement. A list of issues was maintained and updated after each session, and issues were prioritized in accordance with the number of users experiencing each problem. Table 1 summarizes the phases of testing.

**Table 1:** Testing phases

Phase	Time period	Users	Main purpose
Testing of first version	Aug 2014 – Jun 2015	20 laypeople, 8 patients, 6 psychiatrists.	Test and improve the generic and the disease-specific features of the system
Testing first redesigned version with added features	Sep 2015 – Dec 2015	19 laypeople, 13 patients, 2 nurses, 3 physicians/psychiatrists.	Assess results of first redesign, test added features, and find remaining and new issues. Summative evaluation.
Testing of second and third redesign, and added features	Mar 2016 – Aug 2016	2 patient-physician dyads, 3 nurses.	Assess results of second and third redesign, find remaining and new issues.

Testing phases until August 2016. Three major redesigns were implemented based on usability tests

#### System usability scale

The SUS scale allows evaluation of platforms and software [47] and is considered an industry standard for measuring usability [43, 48, 49]. The average SUS score for 500 products is 68 [43]; a SUS score above this level is often referred to as a “good” product, whereas a score above 85 is considered “excellent” [48, 50].

#### Summative usability testing

In summative usability testing, a product is quantitatively evaluated with representative users and tasks to measure usability, efficiency, and satisfaction [43, 51]. We piloted the summative test with seven patients and then clarified the instructions and tasks given. Eight scenarios and tasks with expected completion times identical to those of a healthy, advanced internet user were presented to five patients. Task completion rate, numbers, and types of errors were measured. Each participant completed a questionnaire (Additional file 4) and the SUS scale (Additional file 5).

#### Long-term pilot

Two doctor-patient dyads tested the system for four weeks. Participants provided SUS scores three times and answered a questionnaire developed by the researchers (Additional file 4).

### Statistical methods and analysis

Standard descriptive statistical methods were used to describe the participants’ demographics and to summarize the data. SUS scores were calculated according to the SUS scoring manual [48]. To compare SUS scores between the different user groups, a one-way ANOVA test was used (IBM SPSS Statistics 22.0, NY, USA).

### Comparative assessment

**Fig. 1:**
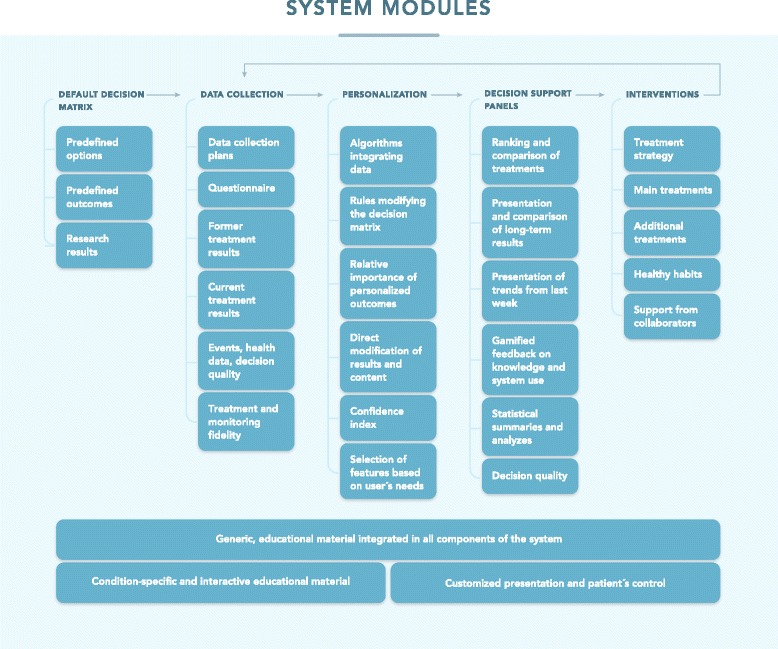
System modules. Simplified overview of the system as per June 2017 from end users’ perspective as a loop of collecting and personalizing information, then using decision support panels to gauge the results and select interventions. The results of those interventions are then collected and a new loop starts

Two authors (KN and ØE) identified modules in the system mathematically integrated by the MCDA-based algorithm and modules providing support for *n* = 1 trials. The modules in the system (Fig. 1) were compared to the AGREE and IPDAS requirements for clinical practice guidelines and patient decision aids, respectively (Table 2).

**Table 2:** Comparison to clinical practice guidelines and patient decision aids

Feature	System	AGREE	IPDAS
Predefined treatment options	x	x	x
Predefined patient-important criteria	x	x^a^	x^a^
Expected performances for all options on all criteria	x	0^b^	x
Patient’s relative preferences for the outcomes, integrated in the mathematical calculation of expected values	x	0	0^c^
Data collection plans	x	0	0
Notifications and reminders on smartphone	x	0	0
Collection of former treatment results	x	0	0
Collection of current treatment results	x	0	0
Collection of life events, health data and decision quality	x	0	0
Measurement of treatment and monitoring fidelity	x	0	0
Automatic modification of core decision components based on patient characteristics	x	0	0
The priorities of the individual patient, and relevant data from the patient, clinician and research, integrated mathematically to provide a ranking of treatments	x	0	0
Direct modification of core decision components available	x	0	0
Ranking of treatments based on mathematical integration includes uncertainty or quality of the evidence assessments	x	0^d^	0^d^
Comparison of expected performance of options	x	0	x
Expected performance of all options provided	x	x	x
Visualization of individual patient results over time	x	0	0
Statistical summaries and analyzes for the individual	x	0	0
Treatment strategy for the individual patient available	x	0	0
Agreed, main treatments for the individual accessible	x	0	0
Agreed, additional treatments for the individual accessible	x	0	0
Agreed, healthy habits for the individual accessible	x	0	0
Support for help from collaborators integrated in system	x	0	0

Comparison of core features in the health optimization system to the requirements in the AGREE and IPDAS standards for clinical practice guidelines and patient decision aids, respectively

^a^ According to AGREE, the benefits and risks should be considered but they do not have to be patient-important. According to IPDAS, criteria have to be provided but it is not explicitly stated that they have to be patient-important

^b^ Evidence should be searched systematically in general

^c^ Patients should be asked to consider which positive and negative features matter most

^d^ Uncertainty is included in the criteria but mathemathical integration is not a requirement

## Results

### Description of the system

**Fig. 2:**
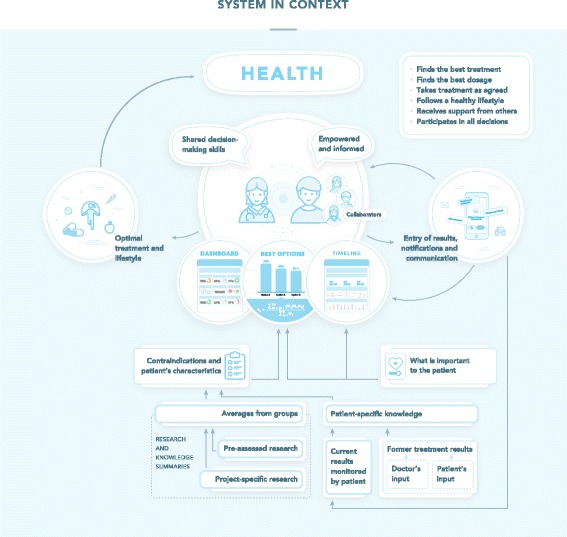
System in context. Overview of system use in optimizing the patient’s health as per June 2017. Patients collect data on their smartphones; these data are integrated with default data from research. The patient and doctor use three types of decision support panels presenting the processed data to make informed decisions on health-promoting interventions together

Fig. 2 presents the health-optimization system in context.

#### Functionality

In the *authoring suite*, authors and co-authors invited by authors can create systems for any long-term condition, for instance bipolar disorder. Authors can tailor the system using different functionalities available in the suite. This condition-specific system is personalized by the individual patient and/or the clinician on the personal website. In the *smartphone app*, patients enter data about their health state and compliance with interventions and can receive reminders for adhering to medicines and lifestyle measures. On the *website*, patients and clinicians can inspect decision support panels and statistics summarising and visualizing the patient’s data, integrated with research, personalized according to the patient’s personal priorities, and adjusted for uncertainty (Fig. 2, Additional file 6). Healthcare personnel and other collaborators can be invited by the patient to use the system and contribute according to rights granted by the patient. Table 3 presents the main features of the system.

**Table 3:** Main optimization strategies and system features

Strategy 1: Find the best treatment
Ranks all available treatments based on all available information and the patient’s preferences.
Allows patients to see how modifying their personal preferences influence this ranking
Presents head-to-head comparisons of all treatments on all included outcomes
Removes treatments that are contraindicated for the specific patient automatically
Removes outcomes that are irrelevant for the specific patient, automatically
Integrates quantitative estimates of treatment effects from research, the patient, and the clinician
Presents treatment results longitudinally together with treatment details and other relevant data
Strategy 2: Find the best dosage
Presents the effects of different dosages on subjective and objective health outcomes as graphs and statistics
Presents the effects of different dosages as an integrated part of complete treatment plans
Strategy 3: Increase treatment adherence
Can remind the patient to take the treatment at all agreed times, on the smartphone
Presents actual use over time as graphs and statistics for patients and clinicians to inspect together
Includes snippets for day-to-day improvement of treatment, lifestyle and monitoring adherence, based on information from the last two weeks
Strategy 4: Live more healthily
Allows patients to select suggested lifestyle measures and include them in the overall treatment plan
Can remind the patient to follow up the lifestyle measures
Presents the adherence to the lifestyle measures graphically and allows inspection of their effects on subjective and objective health outcomes
Allows inspection of how lifestyle, defined as significant events added into the system, affects health, in graphs and from statistics
Strategy 5: Get support from healthcare providers, friends and family
Enables the patient to give healthcare provideres, friends and family access to the patient’s system
Enables the patient to set rules regarding when others should be warned, for instance when adherence has dropped below a pre-defined level
Strategy 6: Improve the decision process and decision satisfaction
Provides information about why and how to be involved in decisions
Enables patients to track the decision quality related to each specific healthcare provider on several aspects
Enables patients and clinicians to make decisions based on patient-specific information integrated with information from research, condensed into graphics and statistics.

Six health optimization strategies are supported by 21 features

In chronic conditions with a strong evidence base, all modules in the system are potentially relevant (Additional file 7).

Additional file 8 provides examples of user interfaces.

#### Textual and numerical descriptions

MCDA prescribes that to make a decision, decision-makers need information about which options are available and which outcomes are relevant, as well as descriptions of the options and outcomes and the expected performance of all options on all outcomes. Overall, 143 dedicated pages containing numerical and textual descriptions of 17 treatment options, 7 outcomes, and 119 ratings were developed. Sixty additional pages present the condition, decision, shared decision-making, and theoretical frameworks. Users can switch between an English and a Norwegian version.

### Results from the formative usability tests

Formative usability tests with a total of 69 participants were performed between October 2014 and July 2016 (Additional file 9). In all, 82 usability issues of varying scope and importance were identified (Additional file 10). Overall, 52% of all issues regarded layout and 28% information delivery. SUS scores collected during this period are presented in Additional file 9.

Feedback on the first version resulted in three major redesigns.

Patients were not satisfied with the system being limited to optimization of medication and wanted a more complete disease management system. The patients’ demands led to the development of adherence support, functionality for selecting and monitoring lifestyle measures, the possibility to enter future therapy and support group appointments, and easy access to their detailed treatment plan.

By late 2016, it became clear that many patients felt overwhelmed when first using the site because of the new functionalities. After redesign, features are no longer presented to the patient unless selected specifically.

As of May 2017, more than 3,600 improvements and bug fixes have been implemented.

### Results from the summative usability tests

All participants (*n* = 5) were women, and the mean (± SD) age was 38 (±7) years. Three had bipolar disorder type I, one had bipolar disorder type II, and one did not know the subtype. All patients had completed at least 12 years of education and reported that their internet literacy was good or very good. The mean ± SD (median) SUS score from the summative test was 78 ± 18 (75). With one exception, all participants completed all tasks on time. Participants committed 0 errors during the 37 tasks completed overall. All the participants strongly agreed that the system helped them to find the best treatment option most accordant with their preferences.

### Mean system usability score

**Fig. 3:**
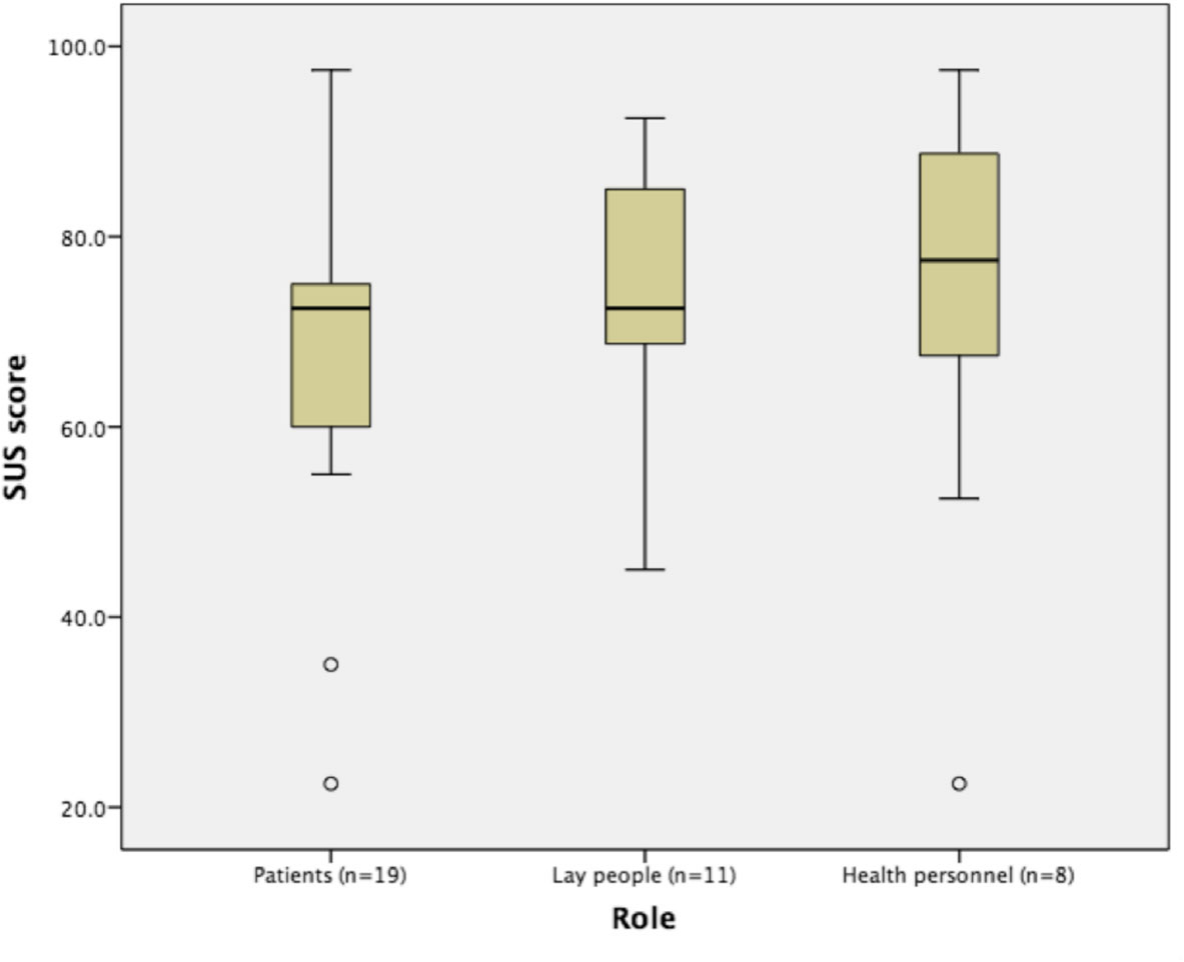
Boxplot of System Usability Scale scores based on roles. Median system usability scores for patients, laypeople and healthcare personnel

SUS scores were collected from 19 patients, 11 laypeople, and 8 healthcare providers. The mean ± SD (median) SUS score for all usability tests performed in 2015 and 2016 was 71 ± 18 (73). Fig. 3 presents the median SUS scores per user group. The SUS scores did not differ significantly between groups (*p* = 0.626).

The mean ± SD (median) SUS score from the summative test was 78 ± 18 (75), suggesting above-average usability and a “good product” [48].

### Piloting of formative, long-term usability testing

One doctor-patient dyad in general practice and another in an outpatient clinic tested the system for four weeks. A registered nurse (SK) who knew the system well taught patients and doctors how to use the system. During consultations, patients and clinicians used the panels to assess the patient’s state, evaluate medications and their dosages, and reflect on priorities and decisions. The second dyad experienced a firewall problem in the hospital, and an iOS update caused the patient’s app to freeze. Both dyads reported the system to be helpful and were generally positive.

### Qualitative feedback

Participants were generally supportive of the health-optimization system. Patients and physicians were particularly satisfied with the panels enabling comparison of options, the possibility to explore how different importance weights influenced the ranking of options, and the Timeline graphs. Here is a sample of quotes from the formative usability testing:

*“Brilliant tool. I especially liked how it improved communication with my doctor” (patient)*

*“The best app I have tested for my condition. This system will be useful for many doctors and patients” (patient)*

*“The system gave our conversation a head start and helped the patient and me concentrate on what was most important to her” (doctor)*

### Comparative assessment

Twenty-two out of the 24 modules in the system could be integrated by means of MCDA-based algorithms (Additional file 7). This finding resonated with the authors’ experience that the MCDA framework accommodated and helped resolve most issues that appeared during development.

Thirteen of the 24 main modules were relevant for *n* = 1 trials in everyday clinical practice (Additional file 7). As the effects of treatments in bipolar disorder can take months or years to establish, graphs can be displayed for different time periods. Statistical summaries of results and monitoring fidelity are provided for all treatment plans (Additional file 8).

The AGREE and IPDAS quality criteria for clinical practice guidelines and patient decision aids together require only 5 out of 23 core features in the health-optimization system (Table 2).

## Discussion

### Principal findings

We have developed a shared digital platform equipping people with bipolar disorder and their clinicians and caretakers, with 21 features supporting the selection of and adherence to health-optimizing interventions. To the best of our knowledge, the system is the first MCDA-based patient and clinician decision aid that integrates longitudinal patient data. The feedback from patients and clinicians has been positive, with high satisfaction levels and perception of usefulness.

The system now includes support for communication and coordination, facilitation of timely decisions, support for the patient’s self-care, and improved compliance [52]. SUS scores and qualitative feedback from patients and clinicians indicate that it might be feasible for patients and clinicians to use the system to collaborate in optimizing treatment.

### Results in context

Like the health-optimization system, clinical practice guidelines and patient decision aids aim to support evidence-based practice, commonly defined as “making decisions about how to promote health or provide care by integrating the best available evidence with practitioner expertise and other resources and with the characteristics, state, needs, values, and preferences of those who will be affected” [53]. However, the three systems differ significantly; the vast majority of components in the health-optimization system are not part of internationally agreed upon requirements for these two genres.

For instance, clinical practice guidelines and patient decision aids do not integrate patients’ weighting of outcomes with estimated treatment effects into a mathematically valid ranking of treatments presenting their expected value for the individual. Although a ranking of treatments is often provided in guidelines, this order is not personalized to the individual based on his or her individual characteristics and weighting of outcomes (preferences). Patient decision aids generally do not provide a ranking; rather, they leave the integration of these preferences and the estimated treatment effects to the cognitive abilities of the patient and clinician, with no patient-specific data. The system sharply contrasts with both approaches; integrating all available information into an overall expected value score.

The health-optimization system differs from traditional patient decision aids in additional regards. First, patient preferences – the relative importance of the outcomes – are elicited to reflect the full and actual range of what to expect from the options [54]. Second, the patient and clinician use identical decision support panels on a common platform. Third, all relevant information from the past and present, from heterogeneous sources, is used to provide a ranking of the treatments.

### Strengths and limitations

Only convenience samples of patients participated in the various parts of the study. The SUS scores concerned different versions and components of the system, were collected at different time-points and should therefore be interpreted with caution.

The number of participants in the summative test was small. Paradoxically, testing with large groups of patients may reduce the quality of the results [43]. The ideal group size for usability testing is debated and depends on the product and the purpose of testing [55]. Usability testing with five people has been found to reveal 80–85% of issues identified in larger groups and has been referred to as a “magic number” [43, 56].

Participants in the study were highly motivated and generally computer-literate. Thus, the generalizability of the usability results to the general patient population, particularly among people with low digital literacy, is uncertain.

### Implications for future research

Many m- and e-health technologies have substantial technical and conceptual shortcomings, limiting their potential as health-optimization technologies. There is therefore a need for innovative systems and for rigorous research evaluations investigating their effects on health outcomes. The same platform and functionalities used to create a system for bipolar disorder are currently being used to develop long-term health-optimization systems for patients with chronic conditions, such as HIV, heart disease and COPD.

## Conclusions

Partly based on current technological genres, we have produce an evidence-based system for the optimal selection of and adherence to interventions in bipolar disorder. The results of feasibility testing are generally positive. If the system is found to improve patient-important outcomes in future research, clinicians might consider prescribing the use of a health-optimization system as a companion to the treatment itself.

### Vailability and requirements

- Project name: A health-optimization system for chronic disorders
- Project home page: https://decidetreatment.org
- Operating system (s): Platform independent
- Programming language: C#, JS
- Other requirements: Windows Server 2012 R2, C# 6.0, EntityFramework 6.1.3, HTML 5, CSS 3.0, web browsers from the past three years.
- License: Prototype license: MIT Expat, Creative Commons Attribution 4.0 International
- Any restrictions to use by non-academics: License needed

## Additional files

Additional file 1: Technical stack. The technical stack used in the development of the system. Overview of technical stack used in the development of the system. (DOCX 15 kb)

Additional file 2: Requirements. Updated. List of requirements for the system. (DOCX 125 kb)

Additional file 3: Description of the system. Description of the system. Textual description of the features in the system. (DOCX 79 kb)

Additional file 4: Questionnaire and answers. SUS scores. The questionnaire used in the summative test and the answers. The answers relate to a 5-points Likert scale where the categories “strongly agree” and “agree”, and the categories “strongly disagree” and disagree”, are aggregated. (DOCX 89 kb)

Additional file 5: System usability scale.doc. pdf,.xls,.txt,.pptx (including name and a URL of an appropriate viewer if format is unusual). The System Usability Scale. The System Usability Scale applied in this study. (DOCX 86 kb)

Additional file 6: Algorithms. Core algorithms used in the system. The formulas used to calculate expected values and confidence indices are provided and explained. (DOCX 126 kb)

Additional file 7: Module uses. Use of the system modules in different contexts. The first figure shows which modules of the system are integrated using MCDA, the second modules relevant in *n* = 1 trials, the third the modules relevant in one-off decisions as opposed to ongoing follow-up, and the forth the modules relevant when the system is used without predefined evidence. (PDF 1978 kb)

Additional file 8: User interface examples. Examples of user interfaces in the system. SUS: system usability scale; SD: standard deviation. Page 1. The patient’s treatment plan, and two panels presenting adherence to medicines and lifestyle measures the last week. Page 2. Panels showing results over time allow patients and clinicians to inspect the results of treatment and a number of variables on the patient’s health. In this example, how the patient is doing is compared to her diary for those days and to alcohol use. The timespan can be up to 1 year. Current treatment and switches in treatment are shown at the top. Page 3. The uppermost panel presents a ranking of treatment options, dynamically modified based on the relative importance of the outcomes entered by the patients. The lower panel allows direct comparison of what to expect from all treatment options, selected from drop-down menus. Page 4. The Statistics page presents all results for any given treatment plan for the time it was in use. (PDF 790 kb)

Additional file 9: Usability tests. An overview of the usability tests. Which parts of the system were tested at what times by whom and with what results. (DOCX 110 kb)

Additional file 10: Usability issues and resolutions. Usability issues and resolutions. Issues identified during the user tests, classified under feature and problem category, and their resolution. Problem categories were adapted from Li et al. Usability testing of ANSWER: a web-based methotrexate decision aid for patients with rheumatoid arthritis. BMC Med Inform Decis Mak. 2013;13:131. (DOCX 34 kb)

## References

[1] Merikangas KR, Jin R, He JP, Kessler RC, Lee S, Sampson NA (2011). Prevalence and correlates of bipolar spectrum disorder in the world mental health survey initiative. Arch Gen Psychiatry.

[2] Gitlin MJ, Miklowitz DJ. The difficult lives of individuals with bipolar disorder: A review of functional outcomes and their implications for treatment. J Affect Disord. 2017;209:147-54. doi:10.1016/j.jad.2016.11.021. 10.1016/j.jad.2016.11.021 PMC7213058 27914248

[3] Geddes JR, Miklowitz DJ. Treatment of bipolar disorder. Lancet. 2013;381(9878):10.1016/S0140-6736(13)60857-0. 10.1016/S0140-6736(13)60857-0 PMC3876031 23663953

[4] Levin JB, Krivenko A, Howland M, Schlachet R, Sajatovic M (2016). Medication adherence in patients with bipolar disorder: a comprehensive review. CNS Drugs.

[5] Vieta E, Colom F. Bipolar disorder in adults: Managing poor adherence to maintenance pharmacotherapy UpToDate: UpToDate; 2016 [updated Jan 09, 2016. Available from: http://www.uptodate.com/contents/bipolar-disorder-in-adults-managing-poor-adherence-to-maintenance-pharmacotherapy.

[6] Yeaw J, Benner JS, Walt JG, Sian S, Smith DB (2009). Comparing adherence and persistence across 6 chronic medication classes. J Manag Care Pharm.

[7] National Institute for Health and Care Excellence (NICE). Medicines optimisation: the safe and effective use of medicines to enable the best possible outcomes (page 7). nice.org.uk: NICE; 2015. Available from: https://www.nice.org.uk/guidance/ng5 26180890

[8] Royal Pharmaceutical Society (RPS) (2013). Medicines optimisation: helping patients to make the most of medicines.

[9] Stacey D, Legare F, Col NF, Bennett CL, Barry MJ, Eden KB (2014). Decision aids for people facing health treatment or screening decisions. Cochrane Database Syst Rev.

[10] Bright TJWA, Dhurjati R, Bristow E, Bastian L, Coeytaux RR, Samsa G, Hasselblad V, Williams JW, Musty MD, Wing L, Kendrick AS, Sanders GD, Lobach D (2012). Effect of clinical decision-support systems: a systematic review. Ann Intern Med.

[11] Blum D, Raj SX, Oberholzer R, Riphagen II, Strasser F, Kaasa S (2015). Computer-based clinical decision support systems and patient-reported outcomes: a systematic review. The patient.

[12] Ranji SR, Rennke S, Wachter RM (2014). Computerised provider order entry combined with clinical decision support systems to improve medication safety: a narrative review. BMJ Qual Saf.

[13] Vervloet M, Linn AJ, van Weert JC, de Bakker DH, Bouvy ML, van Dijk L (2012). The effectiveness of interventions using electronic reminders to improve adherence to chronic medication: a systematic review of the literature. J Am Med Inform Assoc.

[14] Hamine S, Gerth-Guyette E, Faulx D, Green BB, Ginsburg AS (2015). Impact of mHealth chronic disease management on treatment adherence and patient outcomes: a systematic review. J Med Internet Res.

[15] Roshanov PS, Fernandes N, Wilczynski JM, Hemens BJ, You JJ, Handler SM (2013). Features of effective computerised clinical decision support systems: meta-regression of 162 randomised trials. BMJ (Clinical research ed).

[16] Quaglini S, Sacchi L, Lanzola G, Viani N (2015). Personalization and patient involvement in decision support systems: current trends. Yearb Med Inform.

[17] Lobach DF (2013). The road to effective clinical decision support: are we there yet?. BMJ (Clinical research ed).

[18] Council TGM (2013). Good medical practice.

[19] Medical Council of New Zealand: Good prescribing practice2015 May 4, 2016; (November 2015). Available from: https://www.mcnz.org.nz/assets/News-and-Publications/Statement-on-good-prescribing-practice-v3.pdf.

[20] Ketter TA (2010). Strategies for monitoring outcomes in patients with bipolar disorder. Prim Care Companion J Clin Psychiatry.

[21] Liu NH, Daumit GL, Dua T, Aquila R, Charlson F, Cuijpers P (2017). Excess mortality in persons with severe mental disorders: a multilevel intervention framework and priorities for clinical practice, policy and research agendas. World Psychiatry.

[22] Makoul G, Clayman ML (2006). An integrative model of shared decision making in medical encounters. Patient Educ Couns.

[23] The Norwegain Ministry of Health. National plan for health and hospitals in Norway 2016-2019; 2015. https://www.regjeringen.no/no/dokumenter/meld.-st.-11-20152016/id2462047/.

[24] Elwyn G, O’Connor A, Stacey D, Volk R, Edwards A, Coulter A (2006). Developing a quality criteria framework for patient decision aids: online international Delphi consensus process. BMJ (Clinical research ed).

[25] IPDAS. International Patient Decision Aid Standards (IPDAS) Quality Checklist: International Patient Decision Aid Standards (IPDAS) Collaboration; 2013 [updated 2013-10-03]. Available from: http://ipdas.ohri.ca/IPDAS_checklist.pdf IPDAS Quality Checklist.pdf.

[26] Thokala P, Devlin N, Marsh K, Baltussen R, Boysen M, Kalo Z (2016). Multiple criteria decision analysis for health care decision making-an introduction: report 1 of the ISPOR MCDA emerging good practices task force. Value Health.

[27] Adunlin G, Diaby V, Xiao H (2015). Application of multicriteria decision analysis in health care: a systematic review and bibliometric analysis. Health Expect.

[28] Dowie J, Kjer Kaltoft M, Salkeld G, Cunich M (2015). Towards generic online multicriteria decision support in patient-centred health care. Health Expect.

[29] Kaltoft MK, Turner R, Cunich M, Salkeld G, Nielsen JB, Dowie J (2015). Addressing preference heterogeneity in public health policy by combining cluster analysis and multi-criteria decision analysis: proof of method. Health Econ Rev.

[30] Kaltoft MK (2015). Towards improved decision quality in person-centred healthcare: exploring the implications of decision support via multi-criteria decision analysis.

[31] Salkeld G, Cunich M, Dowie J, Howard K, Patel MI, Mann G (2016). The role of Personalised choice in decision support: a randomized controlled trial of an online decision Aid for prostate cancer screening. PLoS One.

[32] Gast DL (2010). Single subject research methodology in behavioral sciences.

[33] Smith JD (2012). Single-case experimental designs: a systematic review of published research and current standards. Psychol Methods.

[34] EVICARE. Evicare - Evidence-based care processes: Integrating knowledge in clinical information systems Cristin.no; 2015. Available from: https://www.cristin.no/app/projects/show.jsf?id=337417.

[35] Eiring Ø, Slaughter L. An Assessment of the Potential for Personalization in Patient Decision Aids. In: Kostkova P, Szomszor M, Fowler D, editors. Electronic Healthcare: 4th International Conference, eHealth 2011, Málaga, Spain, November 21-23, 2011, Revised Selected Papers. Berlin, Heidelberg: Springer Berlin Heidelberg; 2012. p. 51-7.

[36] Eiring Ø, Nylenna M, Nytrøen K (2016). Patient-important outcomes in long-term treatment of bipolar disorder: a mixed methods approach investigating relative preferences and a proposed taxonomy. The patient.

[37] Cunich M, Salkeld G, Dowie J, Henderson J, Bayram C, Britt H (2011). Integrating evidence and individual preferences using a web-based multi-criteria decision analytic tool: an application to prostate cancer screening. The patient.

[38] Dolan JG, Veazie PJ, Russ AJ (2013). Development and initial evaluation of a treatment decision dashboard. BMC Med Inform Decis Mak.

[39] OECD Better Life Index. oecd.org. Available from: http://www.oecdbetterlifeindex.org/.

[40] Eiring Ø. Personalised decision support for patients with bipolar disorder: Research protocol. Oslo: University of Oslo; 2013.

[41] Eiring Ø, Landmark BF, Aas E, Salkeld G, Nylenna M, Nytrøen K. What matters to patients? A systematic review of preferences for medication-associated outcomes in mental disorders. BMJ Open. 2015;5(4):e007848. doi:10.1136/bmjopen-2015-007848. 10.1136/bmjopen-2015-007848 PMC4390680 25854979

[42] Eiring Ø, et al. Multi-criteria decision analysis of pharmacological maintenance treatment in bipolar disorder: Evaluation of an expedite yet comprehensive approach. [Original research article]. In submission.

[43] Sauro J, Lewis JR (2012). Quantifying the user experience. Practical statistics for user research.

[44] Nemeth CP. Usability assessment. Human Factors Methods for Design: Making Systems Human-Centered. Boca Raton: CRC Press; 2004. https://www.crcpress.com/Human-Factors-Methods-for-Design-Making-Systems-Human-Centered/Nemeth/p/book/9780415297981.

[45] Garrett JJ (2010). Elements of user experience: user-centered design for the Web and beyond (voices that matter).

[46] Krug S (2010). Rocket surgery made easy.

[47] Brooke J, Jordan BT PW, Weerdmeester BA, McClelland AL (1996). SUS: a “quick and dirty” usability scale. Usability evaluation in industry.

[48] Sauro J (2011). A practical guide to the system usability scale: background, benchmarks, and best practices.

[49] System Usability Scale (SUS). usability.gov. Available from: https://www.usability.gov/how-to-and-tools/methods/system-usability-scale.html.

[50] Bangor AKP, Miller J (2009). Determining what individual SUS scores mean: adding an adjective rating scale. J Usability stud.

[51] Summative Usability Testing: usabilitybok.org; 2010. Available from: http://www.usabilitybok.org/summative-usability-testing

[52] Chouvarda IG, Goulis DG, Lambrinoudaki I, Maglaveras N (2015). Connected health and integrated care: toward new models for chronic disease management. Maturitas.

[53] Evidence-Based Behavioral-Practice (EBBP). Bridging Research and Practice: Evidence-Based Behavioral-Practice. Available from: http://www.ebbp.org/

[54] Felli JC, Noel RA, Cavazzoni PA (2009). A multiattribute model for evaluating the benefit-risk profiles of treatment alternatives. Med Decis Making.

[55] Macefield R (2009). How to specify the participant group size for usability studies: a Practitioner’s guide. J Usability Stud.

[56] Virzi RA (1992). Refining the test phase of usability evaluation: How many subjects is enough?. J Hum Factors Ergon Soc.

