# Supplementary material for: The development and feasibility of a personal health-optimization system for people with bipolar disorder
Source: BMC Med Inform Decis Mak. 2017 Jul 10;17:102. doi: 10.1186/s12911-017-0481-x (PMC5504814; doi:10.1186/s12911-017-0481-x)
Supplement: Supplementary file 1 — Technical stack. The technical stack used in the development of the system. Overview of technical stack used in the development of the system. (DOCX 15 kb) [file 12911_2017_481_MOESM1_ESM.docx]

**Appendix 1: Technical stack**

*Back-end:* DECIDE treatment is a client-server web application. The client side is rendered as a web application and a hybride mobile application. The system was constructed and is maintained on Windows Server 2012 R2, using the programming languages C#, JS and the database Azure SQL Server 2016. The software server-side was developed based on the ASP.NET technology with .NET Framework stack, which implements Model View Controller pattern in ASP.NET MVC 5. All system sources are located in Microsoft Azure Cloud. The web application generates all web pages and manages user accounts, and operates Web Cache to achieve faster data accessing. Other requirements are Microsoft Identity 2.0, Microsoft Web Api 2.0, Microsoft Azure, C# 6.0, EntityFramework 6, HTML 5, CSS 3.0, GitHub, Jenkins 1.651.3, JMeter 2.13 and web browsers from the last three years.

*Front-end:* Html 5, CSS 3, JS, SVG. Libraries: D3, jQuery, Bootstrap, Moment, DataTables, unobtrusive validation, set of minor plugins. Handmade solutions, component approach, custom UI.

*QA:* TestRail, Fiddler, Postman, Wireshark, Web Browser DevTools, Firebug, OWASP ZAP, Jmeter, Android SDK Tools, Jenkins, Maven, Selenium WebDriver and TestNG*.* Autotests cover about 30 percent of all tests-cases in the web application.

*Mobile app:* The hybrid mobile application is built with Apache Cordova 5.1.1 framework and PhoneGap distribution in Xcode environment for iOS and Android studio for Android. The minimum technical requirements for the devices are Android 4.1 or iOS 8.0. Components: Apache Cordova, Ionic SDK, set of minor plugins.

*Security*

Cryptographic protocol Transport Layer Security (TLS 1.2.) provides communication security over the computer network. The data in the database are replicated in three different datacenters.

The license to run Windows Server, SQL Server in Azure environment is by default included in the Azure Services. The libraries involved are used according to license agreement of NuGet Gallery.
