# Supplementary material for: The development and feasibility of a personal health-optimization system for people with bipolar disorder
Source: BMC Med Inform Decis Mak. 2017 Jul 10;17:102. doi: 10.1186/s12911-017-0481-x (PMC5504814; doi:10.1186/s12911-017-0481-x)
Supplement: Supplementary file 2 — Requirements. Updated. List of requirements for the system. (DOCX 125 kb) [file 12911_2017_481_MOESM2_ESM.docx]

**Appendix 2: Requirements**

|  | A complete health optimization system |
| --- | --- |
|  |  |
| 1 | A clear presentation of the decisions addressed in the system and the significance of patient participation in these decisions |
| 2 | A clear presentation of how the patient´s priorities affect the ranking of options |
| 3 | A clear presentation of all relevant and available options |
| 4 | Support for manual and automatic personalization of options including removal of contraindicated options |
| 5 | A clear presentation of all main outcomes important to patients |
| 6 | Support for manual and automatic personalization (remove, add) of outcomes |
| 7 | A clear and numerical presentation of what the patient can expect from all options on all outcomes |
| 8 | Support for manual and automatic adjustment of the numerical, expected performance for all options on all outcomes including a separate workflow for entering former treatment results, for patients and doctors. |
| 9 | Support for direct, quantitative comparison of all treatment options on all outcomes |
| 10 | A mechanism and method for patients to trade off outcomes based on expected performances of best and worst option |
| 11 | An algorithm and user interface ranking all options, based on a mathematically valid integration of the patient´s preferences with all available information from the patient, the clinician and research, including uncertainty regarding treatment effects |
| 12 | Mechanisms facilitating patients´ and clinicians´ collaboration and agreement on treatment |
| 13 | Mechanisms facilitating timely support from clinicians, relatives and friends in times of need |
| 14 | A clear presentation of current and all former unique treatment combinations, including dosages, all changes in treatment, lifestyle measures and all non-medical measures |
| 15 | Continuous collection, presentation and analysis of self-reported patient data over time, regarding:  1. Subjective outcomes  2. Adherence  3. Life events, side effects and appointments  4. Objective health data  6. Relative importance of outcomes  7. Decision quality |
| 16 | Integration of data into   1. Total value of treatments and treatment plans 2. Total decision quality 3. Total monitoring fidelity for different time periods |
| 17 | Smartphone support for  1. Easy entry of data from the patient  2. Reminders to take treatment  3. Self-help and lifestyle support |
| 18 | Support for visual inspection of the effects of changes in treatment on patient-important outcomes and other variables, and the effect of life events and health data on the outcomes |
| 19 | Statistical summaries of all relevant variables for each variant of all treatment combinations |
| 20 | E-learning for patients and clinicians teaching the use of and framework behind the system |
| 21 | A notification system enabling information and requests between all collaborators and the patient |
| 22 | Possibilities for the patient to personalize the content, features in use and functionality |
| 23 | Control of user rights and log data for all system use available to the patient |
| 24 | Separate interfaces for patients, clinicians, and other collaborators |
| 25 | Integration of information from different optimization systems when the patients has more than one condition |
| 26 | Aggregation of data from patients for research and quality improvement and mechanisms for improving patients´ decision aids by use of these data |
| 27 | Documentation of all decisions taken and all information provided at time of decision |
| 28 | Integration in the electronic medical journal |
| 29 | “Snippets” presenting the most essential information in each Timeline panel based on data from the last two weeks and tips on how to improve treatment based on these data, on the website and in the app. |
