# Supplementary material for: The development and feasibility of a personal health-optimization system for people with bipolar disorder
Source: BMC Med Inform Decis Mak. 2017 Jul 10;17:102. doi: 10.1186/s12911-017-0481-x (PMC5504814; doi:10.1186/s12911-017-0481-x)
Supplement: Supplementary file 3 — Description of the system. Description of the system. Textual description of the features in the system. (DOCX 79 kb) [file 12911_2017_481_MOESM3_ESM.docx]

**Appendix 3: Description of the system**

DECIDE Treatment is a cloud-based authoring suite enabling the construction of heterogeneous patient-centered decision support systems including health and treatment optimization systems. The platform was used to construct a health optimization system enabling patients with bipolar disorder, their healthcare providers and caretakers to collaborate on the assessment, selection, adjustment and systematic follow-up of treatment use and lifestyle measures, independent of their location.

The system consists of a smartphone app, a web portal for browser-based access, and a server for data storage. Data from the patient, the clinician and research are summarized, integrated and visualized in a series of panels on the patient´s personal webpage. The panels were designed to support and not replace informed discussions between patients and clinicians. Outcomes important to the individual patient form the basis for evaluations of the long-term development of the disease, treatment alternatives, treatment adjustments and treatment effects. The system merges features commonly found in self-monitoring and communication systems with features from patient decision aids, decision support systems for clinicians and chronic disease management applications, into one, integrated system.

The health optimization system for people with bipolar disorder was designed to address situations where the effects of or adherence to the current treatment is unsatisfactory, when patients feel unsure about whether to start, stop or switch treatment, and when it is unclear which treatment or treatment dosage would be most beneficial for the individual. The system can be used intensively, for instance when medication is initiated, switched or adjusted, infrequently, or not at all, for instance when the patient is stable and the effect of the treatment is satisfactory.

The system supports 6 different treatment optimization strategies, each strategy backed by 2 - 7 features:

Strategy 1: Find the best treatment

1. Ranks all available treatments based on all available information and the patient´s preferences.
2. Allows patients to see how modifying their personal preferences influence this ranking
3. Presents head-to-head comparisons of all treatments on all included outcomes
4. Removes treatments that are contraindicated for the specific patient automatically
5. Removes outcomes that are irrelevant for the specific patient, automatically
6. Integrates quantitative estimates of treatment effects from research, the patient, and the clinician
7. Presents treatment results longitudinally together with treatment details and other relevant data

Strategy 2: Find the best dosage

1. Presents the effects of different dosages on subjective and objective health outcomes as graphs and statistics
2. Presents the effects of different dosages as an integrated part of complete treatment plans

Strategy 3: Increase treatment adherence

1. Can remind the patient to take the treatment at all agreed times, on the smartphone
2. Presents actual use over time as graphs and statistics for patients and clinicians to inspect together
3. Includes snippets for day-to-day improvement of treatment, lifestyle and monitoring adherence, based on information from the last two weeks (from March 2017)

Strategy 4: Live more healthily

1. Allows patients to select suggested lifestyle measures and include them in the overall treatment plan
2. Can remind the patient to follow up the lifestyle measures
3. Presents the adherence to the lifestyle measures graphically and allows inspection of their effects on subjective and objective health outcomes
4. Allows inspection of how lifestyle, defined as significant events added into the system, affects health, in graphs and from statistics

Strategy 5: Get support from healthcare providers, friends and family

1. Enables the patient to give healthcare provideres, friends and family access to the patient´s system
2. Enables the patient to set rules regarding when others should be warned, for instance when adherence has dropped below a pre-defined level

Strategy 6: Improve the decision process and decision satisfaction

1. Provides information about why and how to be involved in decisions
2. Enables patients to track the decision quality related to each specific healthcare provider on several aspects
3. Enables patients and clinicians to make decisions based on patient-specific information integrated with information from research, condensed into graphics and statistics.

The specific features are created to facilitate patient-specific insights that cannot be inferred from research or short consultations that are not backed up by data from the individual. It includes measures for increased safety, such as the possibility for the patient, clinicians and relatives to inspect common contrindiciations, and introduces person-specific, longitudinal results into the consultation.

The first version of the health optimization system included preliminary versions of four of the later seven features supporting the selection of treatment (strategy 1, feature 1 – 3 and 7), and a first version of the interface for physicians and carers (strategy 5, feature 1).

To help patients optimize their treatment and achieve the best possible outcomes from their medicines, the system

1) enables patients to select which outcomes should be monitored and to determine the relative desirability of these outcomes.

2) enables the consideration of determinants affecting the outcomes in addition to medication, such as life events, lab values, adherence and non-medical interventions.

3) enables the evaluation of responses to treatment using single subjects research designs features, such as visual inspection of graphs and identification of graph patterns when treatments are introduced and removed, and statistical analysis.

Smartphone apps for iOS and Android provide input mechanisms for patients to enter data in accordance with their monitoring plans. The app prompts the patient to submit subjective and objective information such as symptoms, functional status, side effects, treatment adherence, objective health data, the relative desirability of the outcomes, events influencing their health, and coming appointments. The app can also remind the user to take the agreed treatment and to follow up on lifestyle measures (Figure 1).

All data are transferred to the personal webpage, accessible for the patient, healthcare providers and caretakers. A back-end server processes the data and condenses the information in panels visualizing the information, either as stylized lines or as bar charts. A dedicated panel presents the current treatment plan: the overall treatment strategy, all medication relevant to the disease, non-medication measures such as psychotherapy, and lifestyle measures. Other panels provide historical overviews of the data directly relevant to treatment decisions: adherence, results of self-assessment of outcomes over time, objective health data, and events. Inserted in the upper part of each of these panels is the treatment or treatment combinations used at any time including dosage, clearly depicting changes in treatment. Additional panels present the development over time of the patient´s assessments of decision quality, the total value of their current treatment, the total value of all treatment plans, the expected total value of all treatment alternatives, and the patient´s relative outcome preferences. In addition, data about treatment effects, treatment use, total value, events, objective health data and monitoring fidelity are also summarized in a dynamically updated statistics report specific for each treatment plan.

The schedule for when to enter data to be collected and displayed is defined in monitoring plans, specifying the frequency and content in the requests for data received by the patient on the smartphone. The plans allow the patient and clinician to select the intensity of monitoring, from none to several times per day. The initial version of the system includes default outcomes and suggests that certain health data measures should be collected, dependent on the condition, but no measurement data are mandatory. When available and entered by authors, the initial version also contains the expected performances of all outcomes on all options. Using a confidence-adjusted weighted sum equation, and expected performance ratings, all treatment options are ranked and compared. The patient and clinician also can explore how the ranking of options is affected by different weightings of the treatment outcomes. Other sensitivity analyses can be performed by disabling criteria and options, and by changing the importance of the confidence index.

The graphs and the dashboard were designed so that users can monitor the most important information at a glance, with minimal cognitive burden. The tool includes essential educational material about the condition, the condition-specific decisions, the “no treatment” alternative, shared decision-making, contraindications and patient-important outcomes. Most labels in the system are clickable, providing a means for users to explore the underlying information, at the same time enabling self-regulation of their exposure to the content and avoiding overload. A description of the theoretical underpinnings, using everyday examples, is included in the system. All content in the system is in English and Norwegian and users can switch language by clicking an icon.

All outcomes are measured on a positively framed 0 to 100 scale enabling mathematical integration of all decision-relevant data and comparison of expected values of current, past and alternative treatments. Health data measurements can be measured on any scale; the user enters an upper and lower numerical limit to define the scale for the variable. The medicine adherence monitoring plan by default is similar to the frequency with which medicines should be taken according to the treatment plan. Notifications are sent to the patient at the agreed time of intake. Significant events in the past, present and future can be entered at any time, in addition the patient can opt-in to be prompted daily to enter events that can influence the outcomes. The patient can receive monthly notifications to re-evaluate their relative weightings of the outcomes, providing a mechanism for reflecting on the priorities over time. Also, the user can select to be asked about decision quality after all changes in the treatment plan. Criteria for assessing decision quality are suggested by the system but can be customized by the patient and relative weightings of decision criteria can be changed at any time. Self-assessment data for one particular point of time can be entered the current or the next day and only once. No retrofitting is possible.

When patients use the system for the first time, a series of “cards” walks the patient through all crucial components of the system and decisions. Contraindicated options and irrelevant outcomes are disabled based on patient characteristics and essential monitoring plans are configured. This configuration can be performed by the patient alone, or it can be done together with a clinician or a decision coach such as a nurse or pharmacist. After completing these linear steps, navigation is self-directed.

Options not included in the system, including medication combinations, can be entered by the patient. Custom criteria can also be added.

Clinicians, coaches and caregivers can access the decision optimization systems if the individual patients have granted such access, from dedicated interfaces for these two groups. The specific rights of these collaborators are also determined by the patient. By default, caretakers have read-only rights. The system is designed for use during and between consultations.

Limited support for patient-clinician communication, for instance clinicians´ suggesting treatments and follow-up plans and patients confirming the plans on their smartphone, ensure collaboration on optimization of medicine use. The system has been embedded in the electronic medical journal of the main EMR provider in Norway.

The system can be used flexibly; as a standalone system used by the patient alone for supporting the treatment, during consultations only, as a base for longitudinal counseling led by a nurse or other member of the clinical care team, or as a support system primarily facilitating collaboration with caregivers and family.

A number of interactive “flows” have been constructed, allowing full personalization, and education of the patient. Not to overload the user, these flows can be completed independently of each other and at a time convenient for the patient. A progress bar presents how many of the flows the patient has completed.


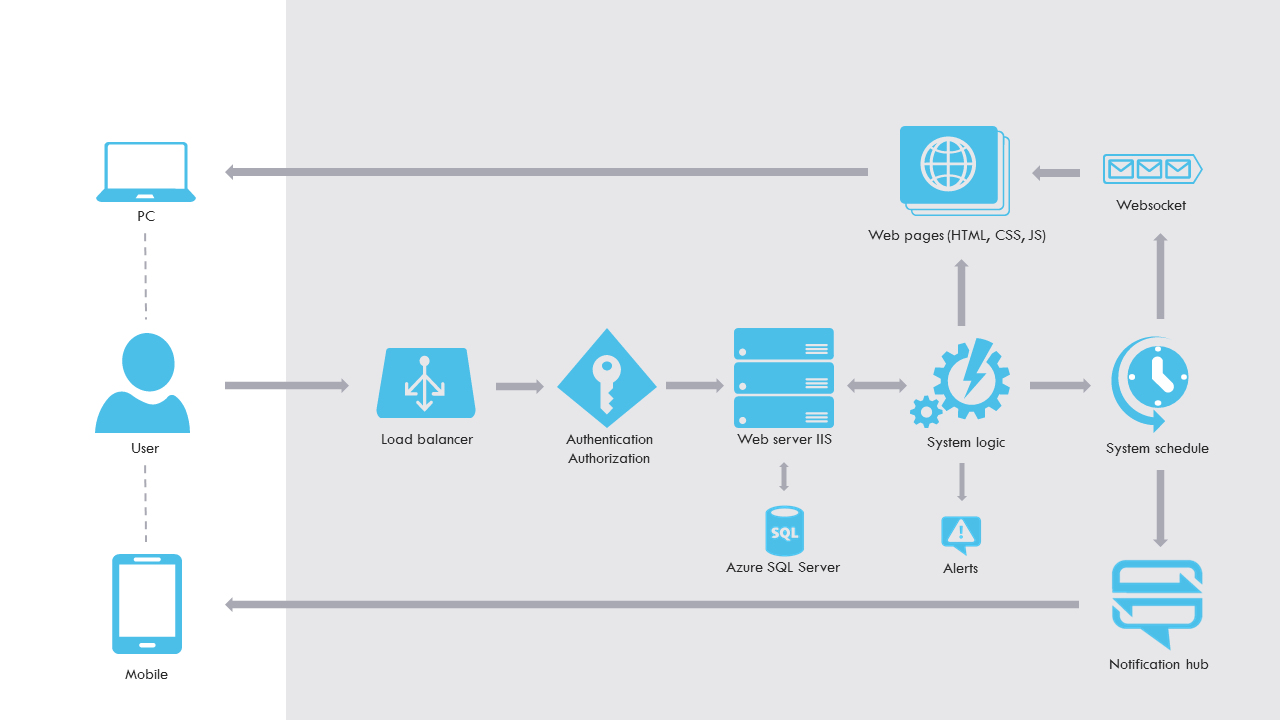


System architecture.
