# Supplementary material for: The development and feasibility of a personal health-optimization system for people with bipolar disorder
Source: BMC Med Inform Decis Mak. 2017 Jul 10;17:102. doi: 10.1186/s12911-017-0481-x (PMC5504814; doi:10.1186/s12911-017-0481-x)
Supplement: Supplementary file 4 — Questionnaire and answers. SUS scores. The questionnaire used in the summative test and the answers. The answers relate to a 5-points Likert scale where the categories “strongly agree” and “agree”, and the categories “strongly disagree” and disagree”, are aggregated. (DOCX 89 kb) [file 12911_2017_481_MOESM4_ESM.docx]

**Appendix 4: Questionnaire and answers**

| **Statement** | **Agree/disagree: percentage N=5** |
| --- | --- |
| 1) After using the system I know more about what the decision regarding long-term treatment in bipolar disorder is about | Strongly disagree / disagree: 0  Neutral: 0  Strongly agree / agree: 100 |
| 2) After using the system I am more aware of why my priorities are important when deciding long-term treatment for bipolar disorder | Strongly disagree / disagree: 0  Neutral: 20  Strongly agree / agree: 80 |
| 3) After using the system I know about more treatment options than before. | Strongly disagree / disagree: 0  Neutral: 0  Strongly agree / agree: 100 |
| 4) The outcomes which are used to evaluate the different treatment options cover all main aspects and are meaningful | Strongly disagree / disagree: 0  Neutral: 0  Strongly agree / agree: 100 |
| 5) The system simplifies finding out what I can expect from the different treatment options | Strongly disagree / disagree: 0  Neutral: 20  Strongly agree / agree:: 80 |
| 6) The system clearly shows how my own priorities affects what appears to be the best treatment option for me | Strongly disagree / disagree: 0  Neutral: 0  Strongly agree / agree: 100 |
| 7) I think the system will help me and my doctor find out what is the best treatment option for me | Strongly disagree / disagree: 0  Neutral: 0  Strongly agree / agree: 100 |
| 8) After using the system I feel more confident and ready to participate in decisions regarding my long-term treatment. | Strongly disagree / disagree: 0  Neutral: 20  Strongly agree / agree: 80 |
| 9) Did you go through the recommended e-learning about shared decision making before today´s test? | Yes: 60  No: 40 |
| 10) I am all in all satisfied with my current treatment | Strongly disagree / disagree: 0  Neutral: 60  Strongly agree / agree: 40 |
| 11) I will probably change my treatment without involving my doctor in the decision | Strongly disagree / disagree: 75  Neutral: 25  Strongly agree / agree: 0 |
| 12) I have become more uncertain about which treatment is the right one for me | Strongly disagree / disagree: 25  Neutral: 25  Strongly agree / agree: 50 |
