# Supplementary material for: The development and feasibility of a personal health-optimization system for people with bipolar disorder
Source: BMC Med Inform Decis Mak. 2017 Jul 10;17:102. doi: 10.1186/s12911-017-0481-x (PMC5504814; doi:10.1186/s12911-017-0481-x)
Supplement: Supplementary file 5 — System usability scale.doc. pdf,.xls,.txt,.pptx (including name and a URL of an appropriate viewer if format is unusual). The System Usability Scale. The System Usability Scale applied in this study. (DOCX 86 kb) [file 12911_2017_481_MOESM5_ESM.docx]

## Appendix 5: System Usability Scale

© Digital Equipment Corporation, 1986.

**Strongly disagree**

1. I think that I would like to use this system

frequently

2. I found the system unnecessarily complex

3. I thought the system was easy

to use

4. I think that I would need the

|  |  |  |  |  |
| --- | --- | --- | --- | --- |

1 2 3 4 5

support of a technical person to

be able to use this system

|  |  |  |  |  |
| --- | --- | --- | --- | --- |

1 2 3 4 5

5. I found the various functions in

this system were well integrated

6. I thought there was too much
 inconsistency in this system

|  |  |  |  |  |
| --- | --- | --- | --- | --- |

1 2 3 4 5

7. I would imagine that most people

|  |  |  |  |  |
| --- | --- | --- | --- | --- |

1 2 3 4 5

would learn to use this system

very quickly

8. I found the system very

cumbersome to use

9. I felt very confident using the

system

10. I needed to learn a lot of

things before I could get going

with this system

.

**Strongly agree**

|  |  |  |  |  |
| --- | --- | --- | --- | --- |

1 2 3 4 5

|  |  |  |  |  |
| --- | --- | --- | --- | --- |

1 2 3 4 5

|  |  |  |  |  |
| --- | --- | --- | --- | --- |

1 2 3 4 5

|  |  |  |  |  |
| --- | --- | --- | --- | --- |

1 2 3 4 5

|  |  |  |  |  |
| --- | --- | --- | --- | --- |

1 2 3 4 5

|  |  |  |  |  |
| --- | --- | --- | --- | --- |

1 2 3 4 5
