# Supplementary material for: The development and feasibility of a personal health-optimization system for people with bipolar disorder
Source: BMC Med Inform Decis Mak. 2017 Jul 10;17:102. doi: 10.1186/s12911-017-0481-x (PMC5504814; doi:10.1186/s12911-017-0481-x)
Supplement: Supplementary file 6 — Algorithms. Core algorithms used in the system. The formulas used to calculate expected values and confidence indices are provided and explained. (DOCX 126 kb) [file 12911_2017_481_MOESM6_ESM.docx]

**Appendix 6: Algorithms**

The rating in each of the cells in the decision matrix expresses the expected performance of one option on one criterion. All ratings are positively oriented on a 0 to 100 scale.

**Sources**

The ratings in each cell are retrieved from one of the following sources:

*Research.* Effect estimates in for instance network meta-analyses, meta-analyses and surveys are converted to expected performance ratings on a 0 to 100 scale. When possible, a health optimization system for a specific condition includes default, research-based ratings for all options on all criteria.

*Former and current experiences based on memory.* When a patient starts using the system, the patient and the physician enter the real effects of former and current treatments based on their memory. Their estimates are converted to expected performance ratings on the 0 to 100 scale. The physician and the patient can later change the estimates by direct editing.

*Current experiences based on monitoring.* Patients can monitor real-time effects of their current treatment by entering data on their smartphone when prompted to by notifications. An average performance based on monitoring, converted to a number on the 0 to 100 scale, is first calculated after a minimum time period defined by the author and thereafter after every new entry of data. All calculations of averages utilize all data since the monitoring started.

*Patient preferences.* Patients provide relative importance weights for each of their criteria in a preference elicitation exercise using the principle of swing-weighting and the self-explicated approach method. This exercise is performed during the initial setup of the system by the patient and can be repeated any time later. The relative weights can also be adjusted directly by the patient at all times.

**Confidence index**

Each of the performance ratings in the decision matrix is given a confidence rating on a 0 to 100 scale. The confidence index represents the confidence that the performance rating will be identical with the performance experienced by the patient and incorporates concepts of uncertainty including the quality of the evidence and the credible interval. It is calculated as follows:

*Research – rules of thumb:*

Survey with patients or experts when relevant, eg. estimate of treatment burden: 50.

Research, significant results but low quality of the studies: 60.

Research, significant results and medium quality of the studies: 70.

Research, significant results and high or very high quality of the studies: 80.

Confidence indices for research ratings are entered by the authors of the condition-specific health optimization system. The maximum confidence index for research is 80 because of the inherent uncertainty in predicting the result on an individual level from studies on patient groups.

*Former and current experiences based on memory.*

When entering a performance rating, patients and doctors also enter their confidence in this rating on a 1 – 5 Likert scale. This confidence is converted into a number between 30 (1) and 90 (100) on the 0 to 100 scale. The maximum value of the confidence rating is 90 because of the inherent uncertainty in predicting a future result from estimates based on memory.

*Current experiences based on monitoring.* During monitoring, patients provide the performance rating for their current treatment on each criterion at times specified by them in the monitoring plan. Patients also enter the actual use of their current treatment. Third, the system measures the monitoring fidelity – the percentage of times monitoring data are entered, compared to what was planned for in the monitoring plan. Forth, the times a patient has to use a treatment before the real treatment effect can be estimated for 75 % (minimum) and 100 % (maximum) of all patients, is entered by the author. The confidence index is the average of the averages of monitoring fidelity and actual use on a 0 to 100 scale, times a score between 75 (minimum time) and 100 (maximum time), dependent on the time passed since the minimum time.

*Selection of performance rating in the cell.* When more than one rating is available, the rating with the highest confidence index is selected in the calculation of the option´s confidence-adjusted expected value. When there are no confidence indices, the confidence index is set to 50 by default.

*Integration of performance ratings***.** For each option, a confidence-adjusted expected value utilizing normalised importance weights, confidence indices and performance ratings for all criteria is calculated.

*Documentation.* All performance ratings from all sources, and all confidence indices are stored in the system and available to the patient and doctor for inspection later.

**Formulas**

Confidence-adjusted expected values for all treatment options are calculated according to the equation

$$Ev=\frac{\sum_{i=1, j=1}^{o, c} (W_{ij}*R_{ij}*I_{ij})}{\sum_{j=1}^{p} W_{j}}$$

where:

***Ev*** *–* Confidence-adjusted, expected value, per treatment option

***o*** – Number of options

***c*** – Number of criteria

***Wij*** – Relative importance weights, per criterion (0-100%)

***Rij*** – The rating per each option per each criterion with the highest confidence index (0-100%)

***Iij*** – Highest confidence index per rating for each option on each criterion (0-100%)

The confidence index for ratings based on the patient´s self-monitoring is calculated using

$$Im=\frac{{(F}_{c}+ C)}{2}*\left( Min+ \frac{(T_{t}*(Max-Min))}{T_{a}-T_{min}} \right)$$

where:

Im – Confidence index for monitoring ratings

Fc – Self-monitoring fidelity as compared to the treatment plan (0-100%)

C – Compliance (0-100%)

Tt – Time period the treatment has been taken

Ta – Treatment time before 100 % certainty about treatment effect is achieved

Tmin – Minimum treatment period before average treatment effect is calculated

Max – Maximum index (100%)

Min – Minimum index (75%)
