# Supplementary figures and images for: The development and feasibility of a personal health-optimization system for people with bipolar disorder
Source: BMC Med Inform Decis Mak. 2017 Jul 10;17:102. doi: 10.1186/s12911-017-0481-x (PMC5504814; doi:10.1186/s12911-017-0481-x)

Appendix 7: Module details

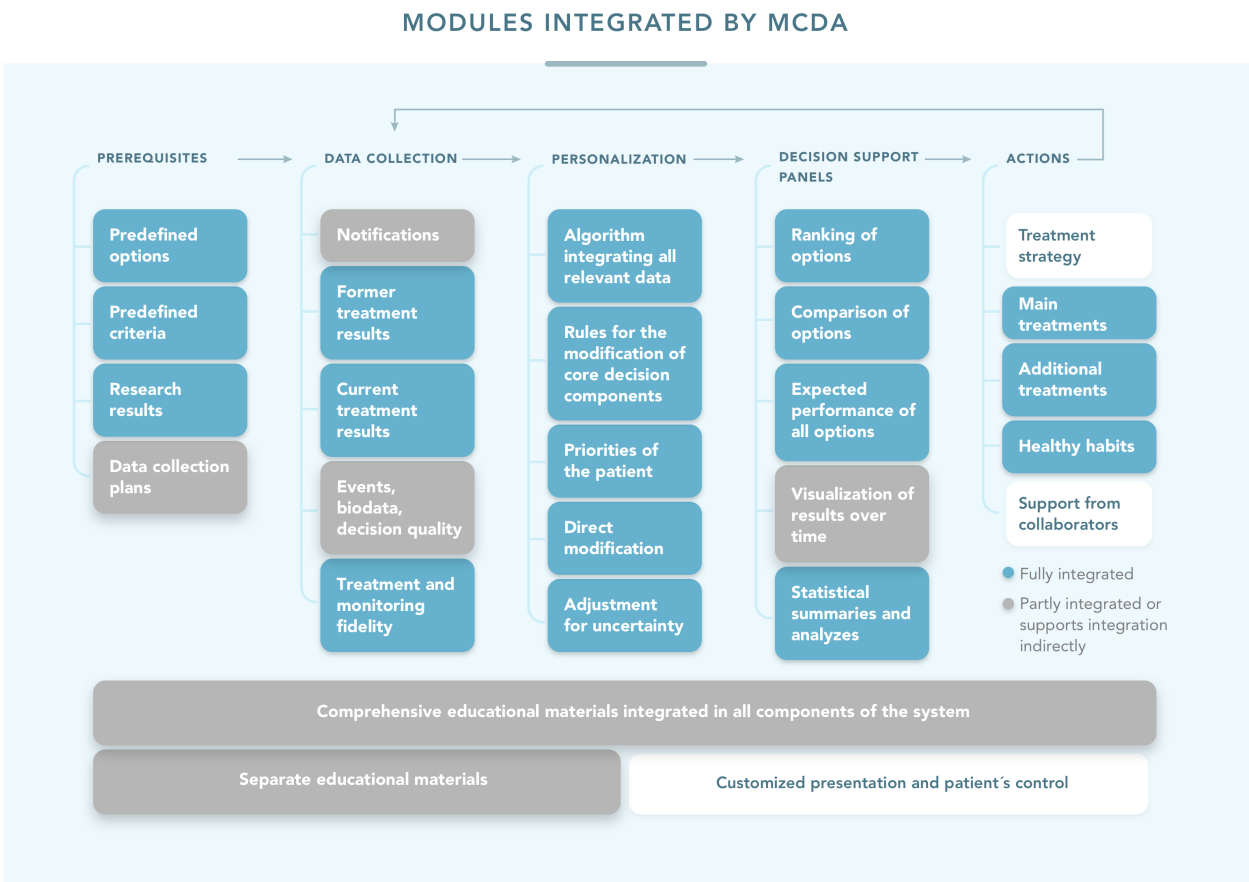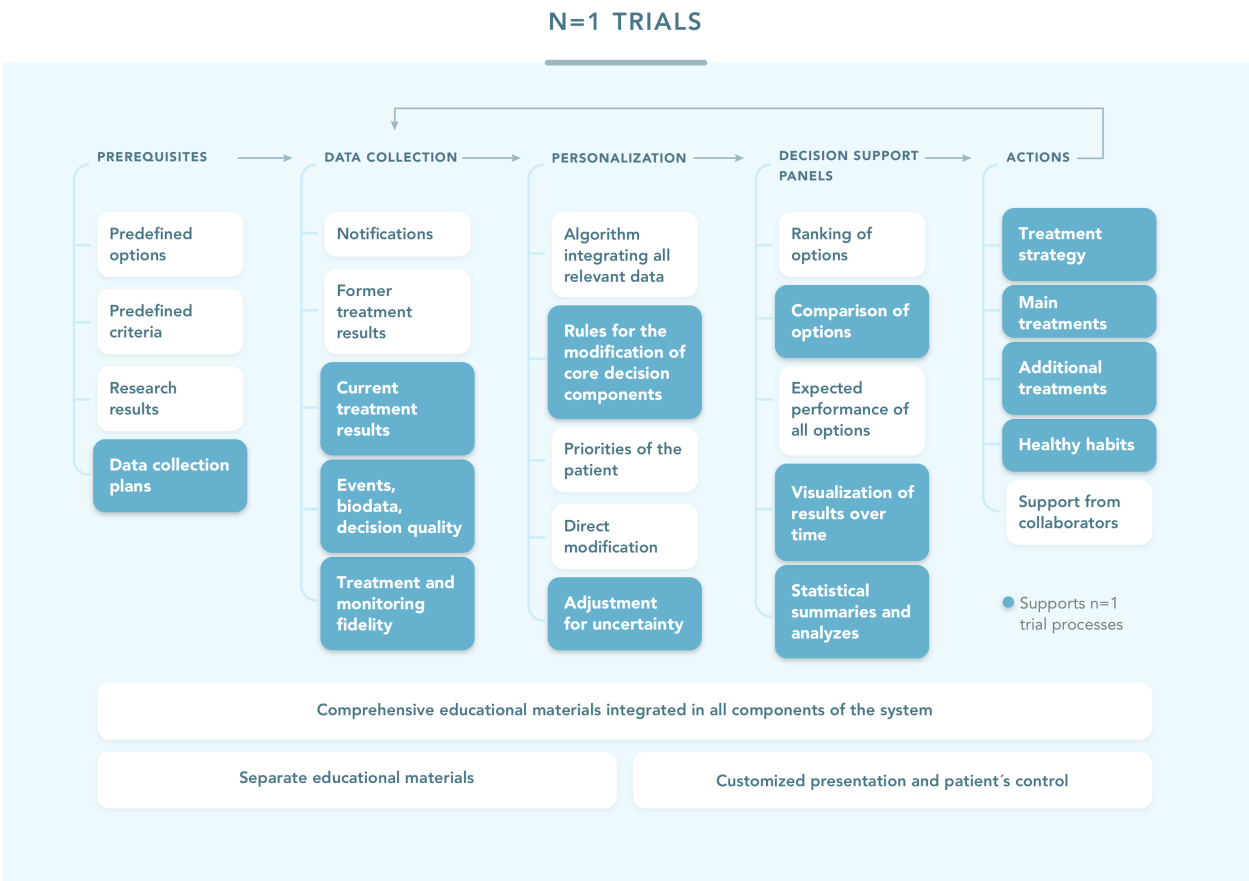

## ONE - OFF DECISIONS

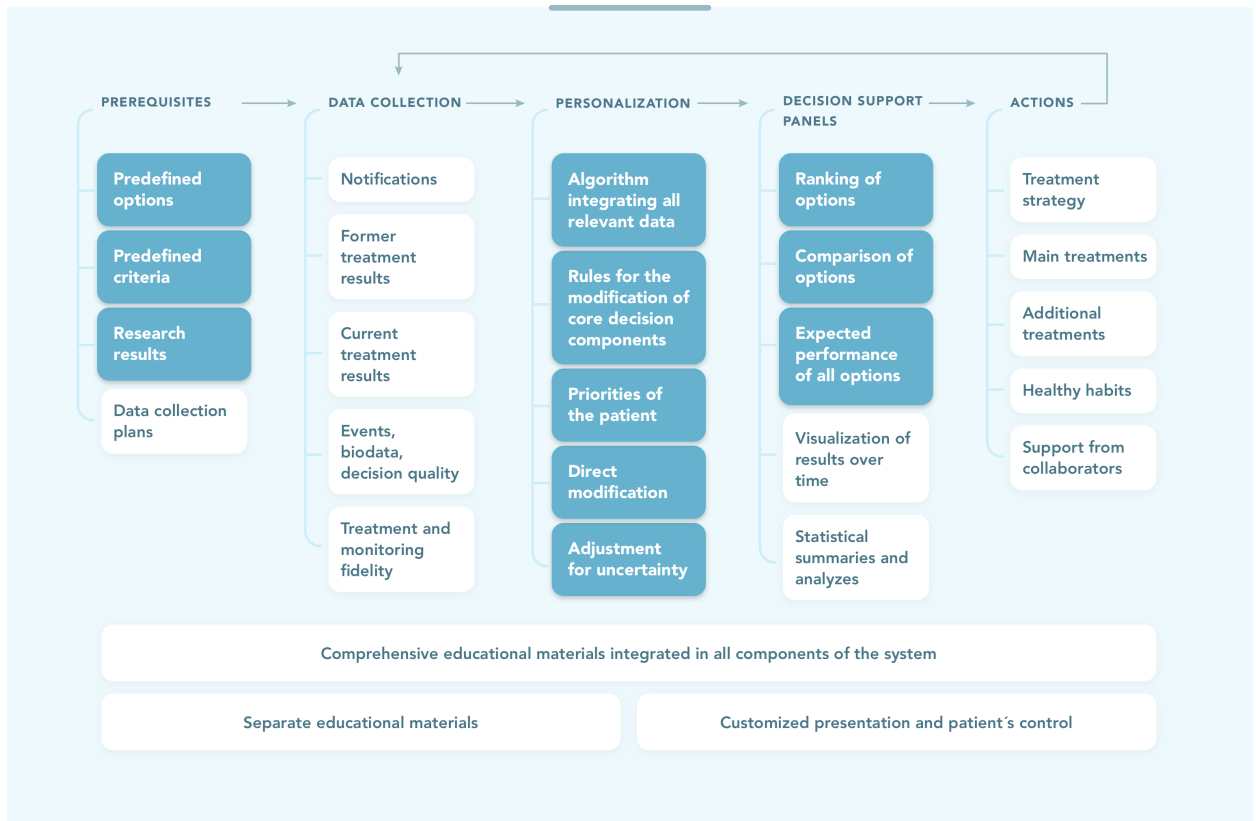

## CHRONIC DISORDERS, NO PREDEFINED EVIDENCE

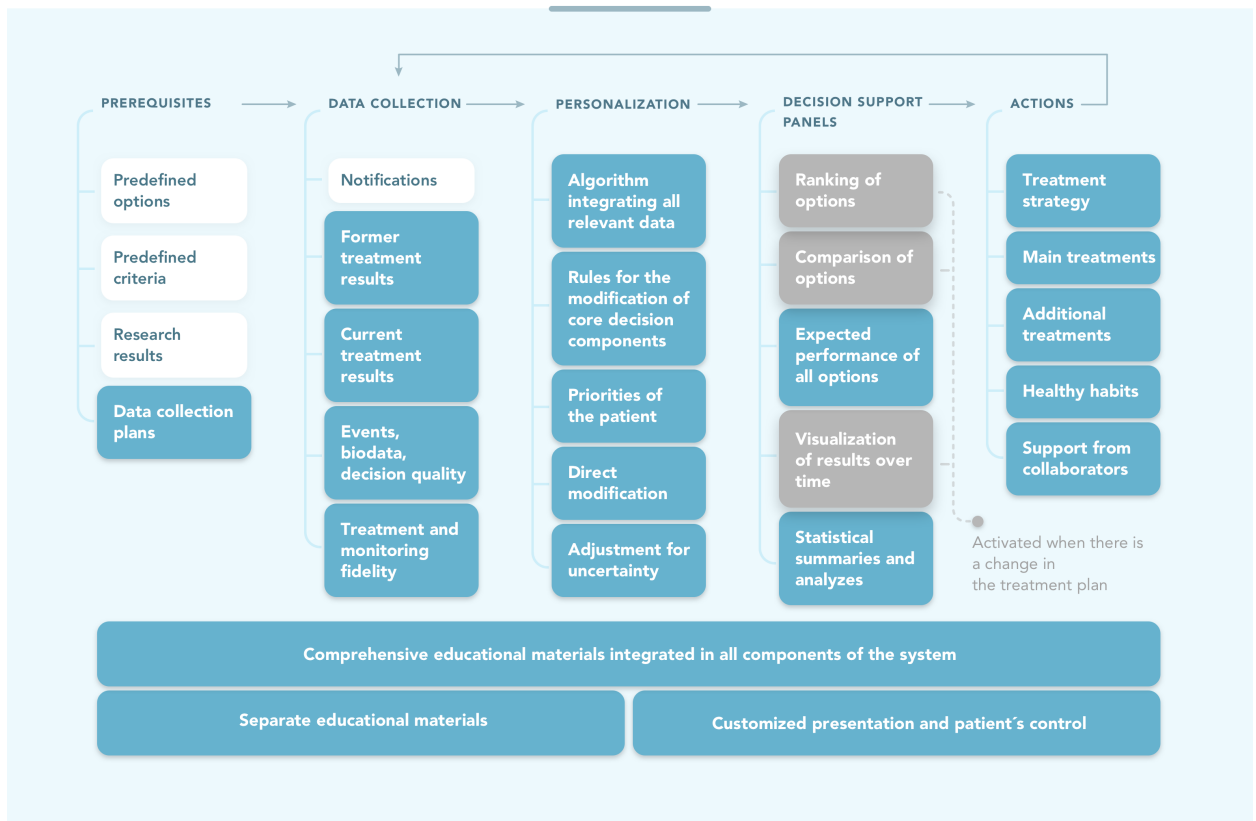

Supplement: Supplementary file 7 — Module uses. Use of the system modules in different contexts. The first figure shows which modules of the system are integrated using MCDA, the second modules relevant in n = 1 trials, the third the modules relevant in one-off decisions as opposed to ongoing follow-up, and the forth the modules relevant when the system is used without predefined evidence. (PDF 1978 kb) [file 12911_2017_481_MOESM7_ESM.pdf]
