# Supplementary material for: The development and feasibility of a personal health-optimization system for people with bipolar disorder
Source: BMC Med Inform Decis Mak. 2017 Jul 10;17:102. doi: 10.1186/s12911-017-0481-x (PMC5504814; doi:10.1186/s12911-017-0481-x)
Supplement: Supplementary file 8 — User interface examples. Examples of user interfaces in the system. SUS: system usability scale; SD: standard deviation. Page 1. The patient’s treatment plan, and two panels presenting adherence to medicines and lifestyle measures the last week. Page 2. Panels showing results over time allow patients and clinicians to inspect the results of treatment and a number of variables on the patient’s health. In this example, how the patient is doing is compared to her diary for those days and to alcohol use. The timespan can be up to 1 year. Current treatment and switches in treatment are shown at the top. Page 3. The uppermost panel presents a ranking of treatment options, dynamically modified based on the relative importance of the outcomes entered by the patients. The lower panel allows direct comparison of what to expect from all treatment options, selected from drop-down menus. Page 4. The Statistics page presents all results for any given treatment plan for the time it was in use. (PDF 790 kb) [file 12911_2017_481_MOESM8_ESM.pdf]

Appendix 9: User interface examples

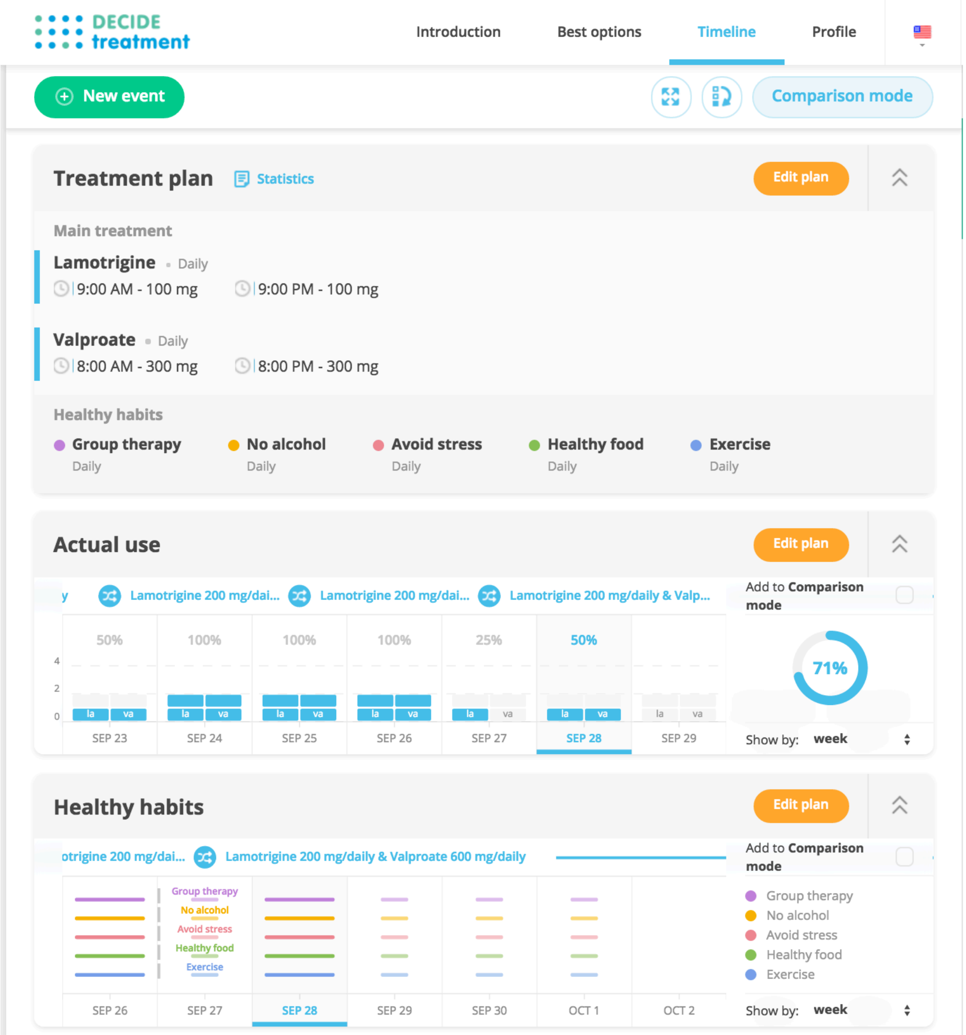

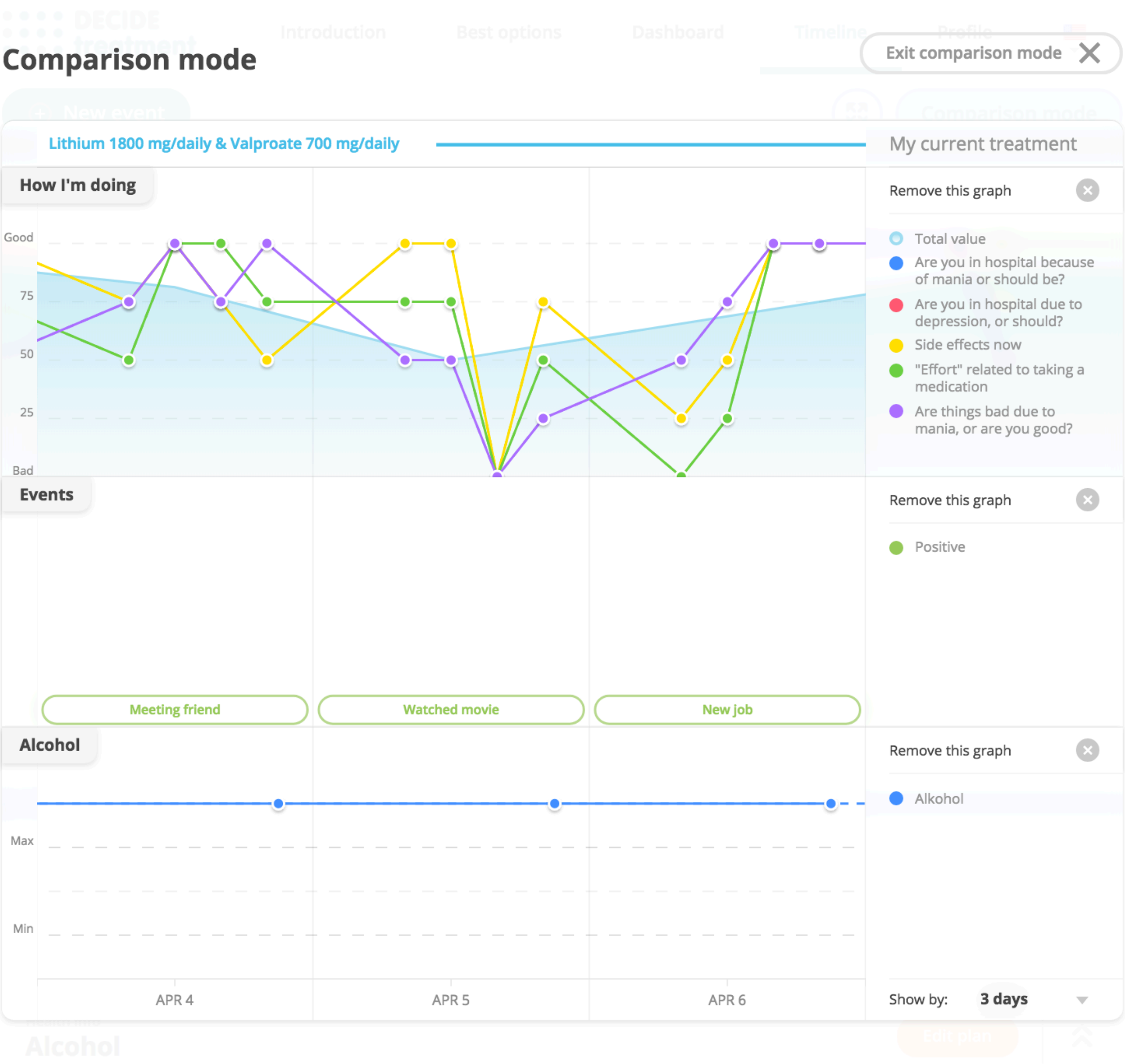
Comparison mode
Exit comparison mode
Lithium 1800 mg/daily & Valproate 700 mg/daily
My current treatment
How I'm doing
Good
75
50
25
Bad
Remove this graph
Total value
Are you in hospital because of mania or should be?
Are you in hospital due to depression, or should?
Side effects now
"Effort" related to taking a medication
Are things bad due to mania, or are you good?
Events
Remove this graph
Positive
Meeting friend
Watched movie
New job
Alcohol
Remove this graph
Alkohol
Max
Min
APR 4
APR 5
APR 6
Show by:
3 days

DECIDE treatment

Introduction | Best options | Timeline | Profile

Best options

Long-term treatment in bipolar disorder

My priorities

Reconsider | Edit priorities

The more important something is to you, the more it counts when the Health Helper calculates the best treatment.

Priority	Scale
Avoid periods with severe mania	Not important – Important
Avoid long periods with severe depression	Not important – Important
Avoid side effects	Not important – Important
Avoid being slightly or quite manic	Not important – Important
Avoid being slightly or quite depressed	Not important – Important

Best options for me

Edit options

Each bar shows how well you can expect the treatment to fit your priorities, all in all.

- 85% Aripiprazole and Lamotrigine
- 82% Lithium and Oxcarbazepine
- 81% Lithium
- 81% Lithium and Valproate
- 79% Lamotrigine
- 77% Valproate
- 59% Lamotrigine and Valproate

The ranking is based on your priorities and the average success rates of the options. Always consult a physician before changing treatment.

Compare options

Aripiprazole and Lamotrigine vs. Lithium and Oxcarbazepine

	Aripiprazole and Lamotrigine	Lithium and Oxcarbazepine
Avoid periods with severe mania	86%	90%
Avoid long periods with severe depression	85%	93%
Avoid side effects	96%	86%
Avoid being slightly or quite manic	80%	66%
Avoid being slightly or quite depressed	74%	54%

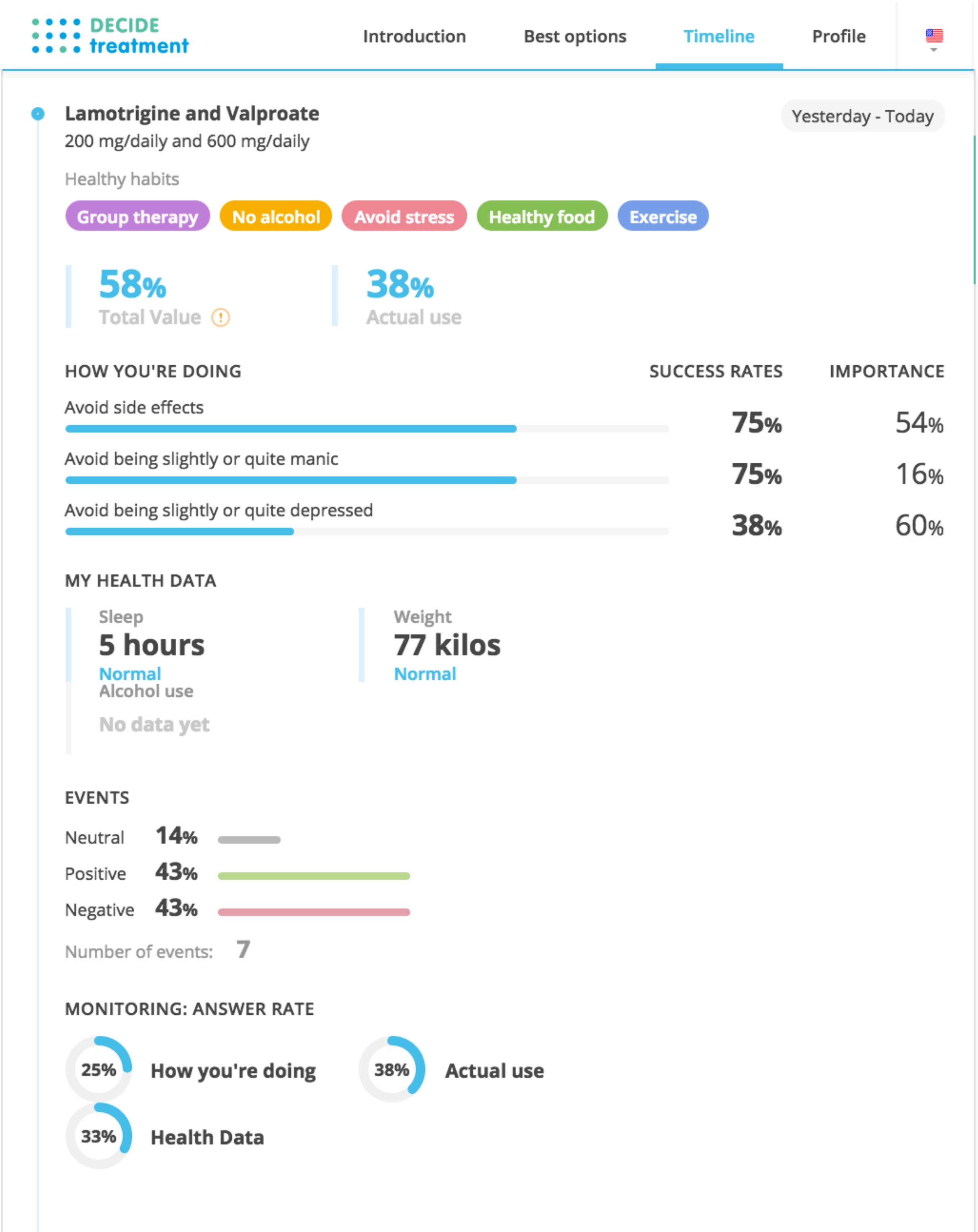
DECIDE treatment
Introduction
Best options
Timeline
Profile
Lamotrigine and Valproate
200 mg/daily and 600 mg/daily
Yesterday - Today
Healthy habits
Group therapy
No alcohol
Avoid stress
Healthy food
Exercise
58%
Total Value
38%
Actual use
HOW YOU'RE DOING
SUCCESS RATES
IMPORTANCE
Avoid side effects 75% 54%
Avoid being slightly or quite manic 75% 16%
Avoid being slightly or quite depressed 38% 60%
MY HEALTH DATA
Sleep
5 hours
Normal
Alcohol use
No data yet
Weight
77 kilos
Normal
EVENTS
Neutral 14%
Positive 43%
Negative 43%
Number of events: 7
MONITORING: ANSWER RATE
25% How you're doing
38% Actual use
33% Health Data
