# Supplementary material for: The development and feasibility of a personal health-optimization system for people with bipolar disorder
Source: BMC Med Inform Decis Mak. 2017 Jul 10;17:102. doi: 10.1186/s12911-017-0481-x (PMC5504814; doi:10.1186/s12911-017-0481-x)
Supplement: Supplementary file 9 — Usability tests. An overview of the usability tests. Which parts of the system were tested at what times by whom and with what results. (DOCX 110 kb) [file 12911_2017_481_MOESM9_ESM.docx]

**Appendix 9: Usability tests**

| **Date** | **Who/what tested** | **N** | **SUS score**  **Mean ± SD (median)** |
| --- | --- | --- | --- |
| **FORMATIVE TESTS (n=69)** | | | |
| Aug 2014 – Feb 2015 | Early formative tests with 20 non-patients | 20 |  |
| Jan 2015 – Jun 2015 | Early formative tests with three patients with bipolar disorder | 3 |  |
| Jan 2015 – Jun 2015 | Early formative tests with six psychiatrists | 6 |  |
| Jun 18, 2015 | Formative test of general functions in the open version of the system (five patients with bipolar disorder) | 5 | 74 ± 14 (73) |
| Sept 1, 2015 | Formative test of general functions in the log-in version of the system with three students (non-patients) | 3 |  |
| Sept 3, 2015 | Formative test of general functions in the log-in version of the system with three students (non-patients) | 3 |  |
| Sept 15, 2015 | Formative test of general functions in the log-in version of the system with three students (non-patients) | 3 | 83 ± 10 (85) |
| Sept 29, 2015 | Formative test of general functions in the log-in version of the system with three students (non-patients) | 3 | 69 ± 11 (70) |
| Oct 10, 2015 | Formative test of general functions in the log-in version of the system with three students (non-patients) | 3 | 74 ± 26 (85) |
| Oct 26, 2015 | One psychiatrists tested the patient´s version and doctor´s version | 1 |  |
| Nov 11, 2015 | Pilot test of the summative usability test with seven patients with bipolar disorder | 7 | 60 ± 25 (71) |
| Nov 16, 2015 | One physician tested the patient´s version and doctor´s version | 1 |  |
| Nov 19, 2015 | Two nurses tested the general functions both in the open – and in the log-in version of the system | 2 | 80 ± 3.5 (80) |
| Dec 1, 2015 | Formative testing of the monitoring functions in the system. Two non-patients used and recorded data in the system daily for three weeks. | 2 | 69 ± 2 (69) |
| Dec 10, 2015 | One doctor (currently undergoing specialization in psychiatry) tested both the patient´s version and the doctor´s version of the system. | 1 | 92 |
| March, 2016 | Formative testing with three nurses: Testing of new initial user flow. | 3 | 68 ± 5 (70) |
| July, 2016 | UK: Landing page, wording (One patient). Ukraine: Best options and Timeline pages (Two non- patients: health technology professionals) | 3 |  |
| **FORMAL, SUMMATIVE TEST (n=5)** | | | |
| Nov 17, 2015 | Summative usability test (open version) with five patients with bipolar disorder | 5 | 78 ±18 (75) |
| **DOCTOR-PATIENT DYAD TESTING (N=2)** | | | |
| April-May 2016 | One doctor/patient dyad tested the full system, including the timeline function for three weeks (score based on 5 different SUS; Patient (2) and doctor (3) | 2 | 72 ± 4 (73) |
| June.August 2016 | One doctor/patient dyad tested the full system, including the timeline function for eight weeks (score based on 5 different SUS); Patient (3) and doctor (2) | 2 | 51 ± 16 (58) |
| Total: | | 78 |  |
