# Supplementary material for: The development and feasibility of a personal health-optimization system for people with bipolar disorder
Source: BMC Med Inform Decis Mak. 2017 Jul 10;17:102. doi: 10.1186/s12911-017-0481-x (PMC5504814; doi:10.1186/s12911-017-0481-x)
Supplement: Supplementary file 10 — Usability issues and resolutions. Usability issues and resolutions. Issues identified during the user tests, classified under feature and problem category, and their resolution. Problem categories were adapted from Li et al. Usability testing of ANSWER: a web-based methotrexate decision aid for patients with rheumatoid arthritis. BMC Med Inform Decis Mak. 2013;13:131. (DOCX 34 kb) [file 12911_2017_481_MOESM10_ESM.docx]

**Appendix 10: Usability issues and resolutions**

| **Feature** | **Problem category** | **Issue** | **Resolution** |
| --- | --- | --- | --- |
| Author part | Layout | Authors of health optimization systems did not understand all features. | The guide for authors was rewritten with examples and more detailed explanations. |
| Author part | Layout | Authors were confused that creation of a health optimization system from a template took a long time. | A text to authors explaining that the creation of a health optimization system from a template could take more than a minute. |
| Best options | Functionality | Users were confused when allowed to weigh criteria that were disabled. | The ability to weigh criteria that were disabled was removed. |
| Best options | Functionality | Users were confused when they found out that options and criteria disabled by the questionnaire could be enabled manually. | Users continue to be able to enable options and criteria disabled by the questionnaire, but a comment was added. |
| Best options | Information delivery | Users did not understand what the last “hint” on the page meant. | The wording in the hint was changed. |
| Best options | Information delivery | Users did not understand what “my ratings” in the decision matrix meant. | The feature was renamed to “Success rates” |
| Best options | Information delivery | Users did not understand what to do when the “Compare options” panel was presented during the initial setup of the system. | The "Compare options" feature was removed from the initial setup of the system. |
| Best options | Information delivery | Physician was concerned that his patient would not know about it when he disabled an option. | Implemented new notification for patient when doctor disabled an option. |
| Best options | Information delivery | Users were confused of what the panel “Compare options” presented. | The explanation text was changed. |
| Best options | Layout | Users clicked on an incorrect icon when instructed to edit their options. | Redesign of the Best options page. |
| Best options | Layout | It was difficult for users to scroll once inside the matrix. | The matrix will be tested again with more users. |
| Best options | Layout | Is was cumbersome for users to scroll to the top of the page every time they wanted to save changes. | The button "Save" is now displayed at the top and at the bottom of the page, so that users can click any of them to save their changes. |
| Best options | Layout | The patient wanted to learn about treatment burden and side effects of an option in the decision matrix, but did not find the descriptions. | The problem was solved during redesign of the matrix, clarifying that each cell in the matrix contains clickable information. |
| Best options | Layout | Users were not able to see the full names of the options and criteria on the Best options page when many vertical bars were presented. | Vertical bars are only shown when there are four options or less, and full names are always displayed. |
| Best options | Layout | “Hints” on the page about how to use it confused some users. | This problem will be tested again with more users. |
| Best options | Layout | Users were confused that options with no expected utility were displayed in the Best options panel. | Options lacking the data necessary to calculate their expected utility are excluded from the Best options panel. |
| Best options | Layout | Users did not find out how to disable a criterion. | The button leading to edit mode of criteria was renamed. |
| Best options | Layout | Users did not understand how to change the relative weight of criteria on the Best options page. | The explanation text was improved and hints about how to use the page added. |
| Best options | Layout | Users were not sure about the meaning of the percentages above the bars on the Best options page. | An explanatory text was added to the page. |
| Decision quality | Functionality | Users did not find the decision quality feature useful in systems constructed for one-off decisions. | Decision quality features were removed from one-off systems. |
| Guide | Information delivery | Users complained that the guide was too long. | The guide was simplified by removing all non-crucial steps and by replacing parts of it with hints. |
| Guide | Information delivery | Users did not understand certain parts of the guide. | Wording in the guide was changed. |
| Guide | Layout | Users found it difficult to close the guide. | The navigation menu in the guide was removed so that a “close” icon is all that remains. |
| Guide | Layout | Users thought that the "guide" button was a menu item. | The position of the button was changed. |
| Guide | Layout | Users did not understand how they could return to a previous step in the guide. | The button name "Previous" was changed to "Back" |
| Guide | Navigation control | Users tried to interact with the system when going through the guide but this was not possible. | Changed settings so that users were allowed to make changes in their system while the guide is open. |
| Guide | Navigation Control | Users were confused when the guide switches to another page in the system. | The guide was split into parts so that there is now one guide for each page, and these guides are not connected. |
| Help panel | Layout | The users struggled to find out how to turn off the help panel. | The help panel was redesigned and the size of the "close" icon increased. |
| Help panel | Layout | Users were confused by the possibility to switch between topics in the help panel. When moving to the next page clicking the arrow at the right, users did not understand where they were or the context of the information. | The help panel was simplified so that it displays only information about clickable items. |
| Initial setup of the system | Information delivery | Users thought that they had to choose something in first step of initial setup, which was not the case. | During redesign of the initial setup, it was clarified that the first step in the setup only contains information about the system. |
| Initial setup of the system | Information delivery | Several users believed the initial user flow was the service itself and did not understand that it was just an introduction to the main site. | An explanation that users see the initial setup only the first time they use the setup, was added. Redesign changed this flow to a stack of “cards” on top of the main site. |
| Initial setup of the system | Information delivery | Users expected to find trade names of the options in the system. | Functionality for automatic display of trade names was added. |
| Initial setup of the system | Information delivery | Criteria settings and editing of the criteria monitoring plan were separated and users did not understand the connection between them. | Criteria settings and editing of the monitoring plan are displayed in succession. |
| Initial setup of the system | Layout | When a user could not continue because of an error in data entry, the system did not scroll the card to the error and the user did not find the error. | Auto-scrolling to the error was implemented. |
| Initial setup of the system | Layout | Users were confused about the meaning of the buttons “Start” and “Continue” in the Monitoring page in the initial setup. | The initial user flow was redesigned and the names of the buttons changed. |
| Initial setup of the system | Layout | Users thought that the system was “tricking” them, because one of the cards in the initial setup of the system contained several cards inside, and the page numbers on these cards were identical. | This problem will be tested again with more users. |
| Initial setup of the system | Navigation control | Users did not find the button “Next” in the questionnaire. | The button was redesigned and moved to a more usual place for this type of button. |
| Initial setup of the system | Navigation control | Users did not understand which button should be clicked to complete the setup. | The initial setup was redesigned. |
| Initial setup of the system | Navigation control | Users were confused when entering the Timeline page after setup of the system because of the lack of data. | This problem will be tested again with more users. |
| Invitation | Information delivery | Users found the text in the email for activating the account confusing. | The text in the email invitations was changed. |
| Landing page | Information delivery | Users had difficulties understanding what the screenshots displayed. | Screenshots from the redesigned site replaced old screenshots and were renamed. |
| Landing page | Layout | It was not obvious to users that the landing page was scrollable and contained more information than initially seen. | A short text and animation encouraging users to scroll to see more information was added to the landing page. |
| Landing page | Layout | It was difficult for users to find essential information about the system on the landing page. | Links in the footer were moved to the top of the landing page and the order of information blocks changed. |
| Monitoring | Functionality | Users were disappointed that it was not possible to enter data for the previous date. | Possibility to enter data for earlier dates is being implemented for certain data types. |
| Monitoring | Information delivery | Users had difficulties using 0-100 sliders for entry of data and the direction from “”worst” to “best”. | The entry of data functionality was redesigned to a 1-5 Likert scale with colour coding and description of each point in the scale. |
| Monitoring | Information delivery | Users did not understand the difference between weighting criteria and the rating of them when entering data. | Different wording of the criteria and different functionality implemented for the two features. |
| Monitoring | Layout | It was difficult for users to set time in monitoring plans and several users clicked on the wrong button. | This problem will be tested again with more users. |
| Monitoring | Layout | Users were confused by the field “comment” when editing monitoring plans. | The “comment” field was removed from monitoring plans. |
| Monitoring | Layout | Users were confused about the difference between “update” and “start” buttons on the same page. | The page was redesigned so that only one button is displayed at the time and the plan is saved by clicking this button. |
| Notifications | Functionality | Users were confused when different times where found for identical notifications on the website and in the mobile app. | Users now see the same time on the website and in the mobile app. |
| Notifications | Information delivery | Users expected the time to take a medication to be displayed in the notification about taking treatment in the mobile app. | The time to take a medication was added to the notification in the mobile app. |
| Notifications | Layout | Users could not find where all current, future and recent notifications were. | This problem will be tested again with more users. |
| Notifications | Layout | Users were confused that different monitoring notifications had the same icon. | Different icons for different monitoring plans are being implemented. |
| Profile | Layout | It was difficult for users to set the date of birth using a datepicker. | The datepicker was replaced with a more usual feature for this type of data. |
| Questionnaire | Information delivery | Users that had not used a medication before did not find out how to enter former treatment. | The answer “Not relevant for me” was added to the questionnaire. |
| Registration and login | Layout | No user entered a password accordant with security standards when asked to enter a password. | An explanation about password requirements was added near the password field. |
| The system | Information delivery | Users did not understand the reason certain pages in the system were present. | Labels and descriptions added to each page of the system. |
| The system | Information delivery | Users were confused by the wording on several pages in the system. Nine different issues regarding wording were found. | Difficult terms were replaced with common day expressions. |
| The system | Layout | Users were confused when a question mark appeared while hovering over a label. The question mark indicated more information. | The question mark was replaced with an underscore signifying a link appearing when hovering. |
| The system | Layout | It was difficult to interact with the system using tablets. | A number of improvements were implemented to optimize the system for use on tablets. |
| The system | Layout | Typos in the Norwegian and English versions were found by users. | The typos were corrected. |
| The system | Layout | Users were confused by the footer because the design was different from other parts of the system. | The footer was redesigned. |
| The system | Layout | Users were confused that pop-ups appeared in different ways in different places in the system. | All system pop-ups were remade, so that they look the same and appear in the same place. |
| Timeline | Functionality | Users did not understand several panels in the Timeline, including decision quality. | The graphs are being redesigned. |
| Timeline | Information delivery | Users expected to find predefined units in the treatment plan. | Predefined units are being added to the system. |
| Timeline | Information delivery | Users did not understand where to find monitoring results. | The Timeline was redesigned so that when users start monitoring they see the place where the results will appear. |
| Timeline | Information delivery | Users did not understand the guide on Timeline because all graphs were empty. | Placeholders with examples of data were added. |
| Timeline | Layout | Users left the Timeline when asked to create an event and did not find the “event” button. | The "add event" button was made available from anywhere on the Timeline page. |
| Timeline | Layout | It was difficult for users to find out where to change their current treatment. | Implementation of the Treatment plan feature solved the problem. |
| Timeline | Layout | It was not obvious for users how to remove an event. | The events editing panel was redesigned. |
| Timeline | Layout | Users clicked on the button “+” instead of entering text into a field. | The button "+" was moved away from the input field, and a placeholder with hints was added. |
| Timeline | Layout | It was not obvious for users that they should click on the time-picker instead of the clock icon. | The clock icon was made clickable. |
| Timeline | Layout | The Timeline page was generally not easy to understand and to interact with. | The Timeline page was fully redesigned and improved taking into account users' suggestions. |
| Timeline | Layout | AM and PM is not used in Norway, so Norwegian users had difficulties with setting the time for monitorings. | Different time formats for the Norwegian version of the system was implemented. |

Problems identified in the different parts of the system during user tests:

|  | Best options | Timeline | Guide | Initial setup | Notifica-tions | Moni-  toring | Landing page | Other | Total |
| --- | --- | --- | --- | --- | --- | --- | --- | --- | --- |
| Layout | 10 | 8 | 3 | 3 | 2 | 3 | 2 | 12 | 43 |
| Functionality | 2 | 1 | 0 | 0 | 1 | 1 | 0 | 1 | 6 |
| Information delivery | 5 | 4 | 2 | 5 | 3 | 3 | 1 | 5 | 28 |
| Navigation control | 0 | 0 | 2 | 3 | 0 | 0 | 0 | 0 | 5 |
| Total | 17 | 13 | 7 | 11 | 6 | 7 | 3 | 18 | 82 |
